# Improvement of Oxidative Stability and Antioxidative Capacity of Virgin Olive Oil by Flash Thermal Pretreatment—Optimization Process

**DOI:** 10.3390/foods14152564

**Published:** 2025-07-22

**Authors:** Dubravka Škevin, Sandra Balbino, Mirella Žanetić, Maja Jukić Špika, Olivera Koprivnjak, Katarina Filipan, Marko Obranović, Karla Žanetić, Edina Smajić, Mateo Radić, Magdalena Bunić, Monika Dilber, Klara Kraljić

**Affiliations:** 1 University of Zagreb Faculty of Food Technology and Biotechnology, Pierottijeva 6, 10000 Zagreb, Croatia; sandra.balbino@pbf.unizg.hr (S.B.); katarina.filipan@pbf.unizg.hr (K.F.); marko.obranovic@pbf.unizg.hr (M.O.); mbunic@pbf.hr (M.B.); mdilber@pbf.hr (M.D.); klara.kraljic@pbf.unizg.hr (K.K.); 2 Institute for Adriatic Crops, Put Duilova 11, 21000 Split, Croatia; mirella.zanetic@krs.hr (M.Ž.); maja.jukic.spika@krs.hr (M.J.Š.); 3 Centre of Excellence for Biodiversity and Molecular Plant Breeding, Svetošimunska Cesta 25, 10000 Zagreb, Croatia; 4 University of Rijeka, Faculty of Medicine, Braće Branchetta 20, 51000 Rijeka, Croatia; olivera.koprivnjak@medri.uniri.hr

**Keywords:** virgin olive oil, flash thermal treatment, Croatian olive varieties, oxidative stability, antioxidant capacity, yield, volatiles, phenolics, tocopherols

## Abstract

Flash thermal pretreatment (FTT) is a promising technique for enhancing virgin olive oil (VOO) quality. This study investigated the effects of FTT, both cooling (15–25 °C) and heating (30–40 °C), on phenolics, tocopherols, fatty acid composition, oxidative stability (OSI), antioxidant capacity (AC), and volatile composition in VOOs from three Croatian varieties: Istarska Bjelica, Levantinka, and Oblica. A full factorial experimental design was used with two independent variables: treatment temperature and olive variety. Olive pastes were treated after crushing and before malaxation. Data were evaluated using ANOVA, partial least squares (PLS) regression, and response surface methodology (RSM). Istarska Bjelica showed the highest OSI improvement (+16%) mostly linked to elevated phenolic compounds. Levantinka exhibited moderate responses, with slight OSI and AC declines. Oblica was most sensitive to heating, showing OSI and AC reductions (up to 28%), despite increased oleocanthal and olacein. RSM identified optimal FTT temperatures for each variety: 18.9 °C (Istarska Bjelica), 15.4 °C (Levantinka), and 15.5 °C (Oblica). These findings support variety-specific FTT as an effective strategy to improve VOO functional and sensory quality.

## 1. Introduction

Olive fruits and virgin olive oil (VOO) are emblematic components of the Mediterranean diet, which has gained global recognition and adoption beyond its traditional geographic origins. VOO is not only a primary dietary fat but also a valuable source of bioactive phytochemicals with demonstrated protective effects against a range of chronic diseases, including cancer, cardiovascular and neurodegenerative diseases, and metabolic syndrome [1,2,3,4].

The chemical composition of VOO is significantly influenced by the technological processes employed during its extraction. In accordance with European Union regulations, VOO must be produced exclusively through mechanical methods, including fruit crushing, malaxation, and centrifugal separation. Among these, the crushing and malaxation steps are particularly critical due to their substantial impact on the extraction and transformation of phytochemicals. Modern industrial extraction employs metallic crushers, which generate substantial emulsification of oil and water phases, thereby necessitating a subsequent malaxation step. During malaxation, the breakdown of emulsions and coalescence of oil droplets occur, while biochemical reactions among compounds released from the crushed fruit further influence oil composition. These interactions govern the distribution of triacylglycerols, fatty acids, pigments, polyphenols, and endogenous enzymes between the aqueous and lipid phases, thereby affecting the organoleptic and nutritional qualities of the final oil. The malaxation phase activates endogenous olive enzymes such as pectolytic and hemicellulolytic enzymes, which degrade cell wall structures and enhance oil release. Lipases, phospholipases, and galactolipases catalyze the hydrolysis of triglycerides, phospholipids, and galactolipids, respectively, liberating fatty acids. Concurrently, enzymes involved in the lipoxygenase pathway, including acylhydrolase, lipoxygenase, hydroperoxide lyase, alcohol dehydrogenase, and alcohol acyltransferase, are responsible for the formation of characteristic volatile compounds. Furthermore, phenolic glucosides are hydrolyzed by β-glucosidase and esterase enzymes to form lipophilic aglycones, while complex polyphenols are cleaved into phenolic alcohols and phenolic acids. Additional oxidative and degradative processes further modulate the phenolic profile [5,6,7,8,9,10,11,12]. The activity of these enzymes determines the partitioning of phenolic compounds between oil and water phases and contributes to the sensory variability observed in VOOs. Enzyme activity is influenced by both intrinsic factors, such as the genetic makeup of the olive variety [13], and extrinsic factors, primarily technological parameters during malaxation [14,15,16,17]. The influence of malaxation duration and temperature on enzymatic activity, phenolic composition, and volatile compound formation has been extensively evaluated, highlighting the importance of optimizing malaxation conditions to improve both extraction efficiency and oil quality. It is generally recommended to malaxate the olive paste gently for 30–45 min at moderate temperatures ranging from 20 to 35 °C [16].

Malaxation is typically conducted in stainless steel chambers equipped with a rotating horizontal shaft fitted with blades that slowly stir the paste. Heating is achieved indirectly through the circulation of hot water within a jacketed chamber. However, conventional malaxation systems present several limitations, including uneven temperature distribution, time–temperature variability across the paste mass, inconsistent flow rates, and prolonged processing times [18]. Advancements in food processing technology have stimulated research into alternative methods for enhancing the malaxation process [19]. Innovative technologies such as microwave heating, ultrasound, and pulsed electric fields have demonstrated potential in improving VOO yield and quality [18,20,21,22].

Among emerging innovations in VOO processing, flash thermal treatment (FTT) has shown considerable potential. FTT involves the rapid heating of olive paste immediately after crushing, with the goal of achieving malaxation temperatures while potentially eliminating the need for conventional batch malaxation. Amirante et al. [23] first introduced this approach by incorporating a heat exchanger post-destoning, enabling instantaneous heating of the paste to 27 °C. This method resulted in significant improvements in total phenolic content, oxidative stability, and overall sensory quality of the oil. Subsequent research employing tubular heat exchangers for paste preheating post-crushing reported similar enhancements in phenolic content, malaxation efficiency, and the volatile profile, mainly due to the reduced malaxation time compared to traditional processes [13,24,25]. Tamborrino et al. [26] advanced the FTT concept by designing a novel rotating spiral heat exchanger, which, either independently or in combination with a microwave module, enabled a 50% reduction in malaxation time, increased oil yield and phenolic content, and preserved the integrity of the volatile fraction. Guerrini et al. [27] applied a brief thermal treatment after 30 min of malaxation at 20 °C. Subsequent heating to 27 °C, and 35 °C improved both yield and total phenolic content (TPC), although treatment at 35 °C induced sensory defects without significantly affecting the volatile composition.

The rapid heating of olive paste is particularly advantageous in cooler growing regions, as it enables rapid and continuous thermal conditioning. Conversely, due to earlier harvest times and the effects of climate change, post-crushing olive paste temperatures may exceed 30 °C, conditions known to be detrimental to EVOO quality by reducing phenolic levels and negatively impacting sensory attributes [7,28]. In response, recent studies have explored rapid cooling as an alternative FTT strategy. Veneziani et al. [28] demonstrated that immediate post-crushing cooling significantly enhanced phenolic content across several Italian cultivars, with no adverse effects on oil yield or legal quality parameters, while volatile compound changes were strongly cultivar-dependent. In 2018, the same group [29] showed that cooling treatments increased phenolic levels and modified volatile profiles, particularly elevating aldehydes such as (E)-2-hexenal, with alcohols and esters influenced by cultivar-specific lipoxygenase activity. Their 2021 study [30] further indicated that cooling to 18 °C followed by malaxation at 25 °C led to phenolic increases of up to 44%, while lower malaxation temperatures reduced phenolic content and variably influenced volatile profiles depending on the cultivar. Notably, under these conditions, the Canino cultivar exhibited enhanced “green” sensory attributes, emphasizing the necessity of cultivar-specific thermal processing protocols. Collectively, these findings underscore the critical role of precise temperature management, both heating and cooling, in optimizing the phenolic profile and sensory quality of EVOO. Importantly, olive cultivar plays a fundamental role in the formation and modulation of phenolic and volatile compounds during thermal treatments. As such, many researchers advocate for the development of cultivar-specific malaxation parameters, particularly when implementing innovative processing technologies, in order to optimize the activity of endogenous enzymes and ultimately enhance oil quality [9,13,14,31,32,33].

Despite extensive research on Italian, Spanish, and Greek varieties, there is a lack of scientific data regarding Croatian olive varieties. Croatia contributes approximately 0.22% to European and 0.13% to global olive oil production [34]. Nevertheless, Croatian VOOs are internationally recognized for their high quality [35] which can be attributed to the country’s considerable genetic diversity among varieties, favorable agroecological conditions, and the expertise of its olive oil producers.

In this context, the primary objective of the present study was to investigate the influence of FTT, including both heating and cooling, on the oxidative stability and antioxidative capacity of virgin olive oils (VOOs) derived from three Croatian autochthonous varieties: Istarska Bjelica, Levantinka, and Oblica. Given their key role in determining these properties, the effects of FTT on phenolic and tocopherol content were also assessed, along with impacts on oil yield and volatile profile. The ultimate goal was to determine variety-specific optimal paste temperatures that support the production of VOO with enhanced oxidative stability and antioxidant potential. To the best of our knowledge, this is the first study to systematically evaluate FTT effects on Croatian olive varieties.

## 2. Materials and Methods

### 2.1. Plant Material

For this experiment, olive fruits (*Olea europaea* L.) of the autochthonous Croatian varieties Levantinka and Oblica were harvested by hand in the same orchard in the Dalmatia region (Croatia) in November 2021, while the variety Istarska Bjelica was harvested in the Istria region (Croatia). The local producers of award-winning EVOOs were asked to suggest the optimum degree of ripeness for each of the selected olive varieties. Accordingly, on the basis of the color of the skin and flesh [36], the fruits of the Oblica variety were harvested at a maturity index (MI) of 1.03, Levantinka 1.08, and Istarska Bjelica 0.48. The fruits of all three studied varieties were harvested at the same phenological stage. However, slight differences in the MI were observed, likely due to variations in the rate of skin pigmentation, which is directly associated with the genetic characteristics of each variety [37].

### 2.2. Olive Oil Production

The olives were processed within 48 h from harvest via centrifugal extraction using the Abencor laboratory oil system (mc2, Ingenierias y Sistemas, Seville, Spain), which simulates the industrial process of VOO production. After cleaning and washing, the olives (about 800 g) were crushed in a hammer mill. Immediately after crushing, the olive paste was subjected to flash thermal treatment (FTT) at six different temperatures (15, 20, 25, 30, 35 and 40 °C) for the purpose of FTT optimization. In order to reach temperatures of 15 and 20 °C, the paste was spread in a 1 cm thick layer on a stainless-steel tray and cooled using a shock freezer at −18 °C (Blastchiller ATT05 ATTILA ABB, TECNODOM, Padova, Italy). Temperatures higher than 20 °C were achieved by heating the paste in a closed plastic container in a water bath (SUB Aqua Pro SAP12, Grant Instruments, Amsterdam, The Netherlands). The water temperature was set to 50 °C (in the case of paste temperatures from 25 to 35 °C) or 52 °C (in the case of a paste temperature of 40 °C). All paste temperatures were reached within 10 min, after which kneading was started immediately. The olive paste was kneaded for 40 min in a thermo-beater at a water temperature of 27 °C. The time required to reach the paste temperature of 27 °C was between 7 and 29 min, depending on the initial temperature of the paste.

The liquid phase was separated via vertical centrifugation at 1370× *g* for 90 s and transferred to a glass cylinder, and the oil fraction was measured to calculate the yield. After separation, the oil was additionally purified via centrifugation (Rotina 380R, Hettich, Tuttlingen, Germany) at 5000× *g* for 4 min at 18 °C. The control sample was prepared under the same conditions of crushing, malaxation, and oil extraction as the other samples, with the exception that no FTT was performed. The average temperature of the control sample after crushing was 22 °C. The entire VOO extraction process was conducted in triplicate for each single combination of variety and temperature, including the control VOOs, and each one was stored in 250 mL dark glass bottles under nitrogen at 15–20 °C until analysis.

### 2.3. Basic Quality Parameters

The basic quality parameters were determined according to the standard methods. The peroxide value of the VOOs was determined according to the ISO 3960 method [38], while the determination of the acidity and the spectrophotometric examination in the ultraviolet range were carried out according to the IOC methods [39,40].

### 2.4. Oil Yield

The oil yield (Y), defined as the amount of oil (g) extracted from 100 g of fruit and expressed in % [41], was calculated according to Equation (1):

(1) $$Y(\%)=\frac{V_{oil}\times 0.915}{W_{olives}}\times 100$$

where V_oil_ (mL) is the volume of the extracted oil, 0.915 (g/mL) is the standard density of VOO, and W_olives_ (g) is the mass of the olive paste.

### 2.5. Volatile Components

The volatiles were isolated via solid-phase microextraction using a 2 cm Divinylbenzene/Carboxen/Polydimethylsiloxane (DVB/CAR/PDMS) fiber with a film thickness of 50/30 µm (Supelco, Bellefonte, PA, USA). The content and composition of volatile compounds were determined by gas chromatography/mass spectrometry (GC/MS) according to our previously published method [42] with some minor modifications. A total of 15 mg of the internal standard (0.15% solution of 4-methyl-2-pentanol in freshly refined sunflower oil) was weighed and transferred to a 20 mL vial with a silicone septum along with 10 g of VOO and then placed in a heating block at 40 °C with a magnetic stirrer (Pierce Reacti-Therm Heating/Stirring Module, Artisan, Champaign, IL, USA). After 10 min sample conditioning, the fiber was exposed for 30 min and then immediately desorbed in the injector of the gas chromatograph in splitless mode at 260 °C for 1 min. The samples were analyzed using an Agilent Technologies 8890 GC system coupled with an Agilent Technologies 7000D TQ mass detector. Volatile compounds were separated on an HP-5 column (30 m × 0.25 mm × 0.25 µm; Agilent Technologies, Santa Clara, CA, USA). The oven temperature was maintained at an initial temperature of 30 °C for 3 min and then increased to 150 °C at a rate of 5 °C/min, followed by an increase to 250 °C at 20 °C/min. This final temperature was maintained for a further 5 min. The temperature of the ion source was 250 °C and that of the transfer line was 260 °C. Helium was used as the carrier gas at a constant flow rate of 1.5 mL/min. The mass spectra were recorded in the mass range 50–550. The volatile compounds were identified by comparing their mass spectra with the records in the NIST mass spectra library. For additional conformation, the retention index values calculated after analyzing the n-alkane mixture (C8–C20) under the same chromatographic conditions were compared with the previously reported values. The quantification of the volatile compounds was carried out using the internal standard mentioned above.

### 2.6. Phenolic Compounds

The polyphenolic compounds were determined by HPLC using a DAD (Agilent Technologies HPLC series 1200, Santa Clara, CA, USA). The extraction of the polyphenolic compounds was carried out according to the IOC method [43] of direct extraction of phenolic compounds by means of a methanolic solution. The extracted phenolic compounds were separated by injecting 20 µL onto a C18 column heated to 30 °C (Luna 250 mm × 4.6 mm, 5 µm, 100 Å, Phenomenex, Torrance, CA, USA). A 0.1% formic acid solution in water (mobile phase A) and a 0.1% formic acid solution in methanol (mobile phase B) were used for elution. The following gradient was used at a constant flow rate of 1 mL/min: At the beginning of the analysis, from 0 to 3 min, 10% B; from 3 to 30 min, the percentages changed to 50% B; from 30 to 40 min, the percentages changed to 60% B and in the next 5 min to 100% B; this percentage remained until 50 min; and from 50 to 50.1 min, the percentages returned to their initial values and remained there for another 10 min. The chromatograms were recorded with the DAD at 280 nm. The quantification of the individual phenolic compounds was carried out according to the IOC method mentioned above.

The phenolic compounds were identified by comparing their retention times and UV spectra with those of commercially available standards. Identification of the remaining compounds was performed via UHPLC Q-TOF-MS analysis (Agilent 1290 Infinity II, Santa Clara, CA, USA) using a Kinetex Core–Shell C18 column (150 mm × 4.6 mm, 2.6 µm, 100 Å, Phenomenex, Torrance, CA, USA) heated to 30 °C. The volume of phenolic extract injected was 3 µL. For the separation of the phenolic compounds, the same mobile phases were used as for the HPLC analysis (A—0.1% formic acid solution in water; B—0.1% formic acid solution in methanol) with an adjusted gradient. In the first 3 min, the separation solution consisted of 10% B; from 3 to 15 min, the percentage changed to 50% B; from 15 to 21 min, the percentage changed to 60% B; and in the next 6 min, the percentage changed to 100% B. This percentage remained up until 30 min; from 30 to 30.1 min, the percentages returned to their initial values and remained there for another 5 min to reach equilibrium. A constant flow rate of 0.4 mL/min was used throughout the analysis. Chromatograms were recorded using DAD (at 280 nm) and Q-TOF-MS detectors. Mass spectra were recorded using an Agilent 6550 Series Accurate-Mass-Quadrupole Time-of-Flight (Santa Clara, CA, USA) system in negative ionization mode with *m*/*z* values ranging from 100 to 1600. Nitrogen was used as a sheath and drying gas at a temperature of 250 °C and a flow rate of 11 L/min and 17 L/min, respectively. The nebulizer pressure was 25 psi, capillary potential was 3500 V, and fragmentor voltage was 380 V. MSMS spectra were recorded in the range of *m*/*z* 50–800 with collision energies of 10–30 V. The phenolic compounds were identified by comparing the obtained mass spectra with those previously published. The compounds detected by UHPLC DAD were identified by comparing the retention times with those of UHPLC Q-TOF-MS. A very good correlation was found between the UHPLC and HPLC retention times with a coefficient of determination of R^2^ = 0.997, which was used for the identification of unidentified phenolic compounds determined by the HPLC DAD method (Supplementary Material Tables S1 and S2).

### 2.7. Tocopherol Content

The tocopherol content was determined according to the ISO standard method [44]. A 1% (*w*/*v*) VOO solution in *n*-heptane was injected (20 µL) into an Agilent Technologies 1260 Infinity series HPLC (Santa Clara, CA, USA) equipped with a fluorescence detector and a LiChroCART Silica 60 column (250 mm × 4.6 mm, 5 µm; Merck, Darmstadt, Germany). The separation of tocopherols was performed for 25 min at room temperature via isocratic chromatography with 0.7% 2-propanol in *n*-hexane as the mobile phase. The flow rate of the mobile phase was 0.9 mL/min. The tocopherols were detected at an excitation wavelength of 295 nm and an emission wavelength of 330 nm. The tocopherols were identified by comparing their retention time with the standards, and a standard solution of *α*-tocopherol was used for quantification, so the results are expressed in mg α-tocopherol/kg VOO.

### 2.8. Fatty Acid Composition

Gas chromatography was used to determine the fatty acid composition of the VOO produced. The fatty acid methyl esters were prepared by transesterification with methanol according to ISO 12966-2:2017 [45]. The produced methyl esters were injected into an Agilent Technologies 6890N Network GC system (Santa Clara, CA, USA) using GC parameters that we have previously reported [42]. The fatty acid methyl esters were identified by comparing their retention times with those of commercial standards, and the amount of each fatty acid is expressed as a percentage of total fatty acids.

### 2.9. Oxidative Stability Index

The oxidative stability index (OSI) of VOO was determined as the induction period (IP) using a DSC 214 Polyma Differential Scanning Calorimeter (NETZSCH, Selb, Germany). The OSI was determined using a modified method previously described by Tan et al. [46]. The instrument was calibrated with pure indium, and an empty aluminum pan identical to the one used for the sample was used as a reference. The VOO (4.0 ± 0.5 mg) was weighed into the aluminum pan, hermetically sealed with a pinhole lid, and placed in the sample chamber of the device. The sample was heated to 140 °C under nitrogen (40 mL/min) at a rate of 20 °C/min, followed by an equilibrium period of 5 min under nitrogen to improve the baseline. To determine OSI, the isothermal method was used at the aforementioned temperature and purified oxygen (99.95%) was passed through the sample cell at 100 mL/min. At the end of the run, IP (min) was determined as the intersection of the extrapolated baseline and the tangent to the recorded exotherm.

### 2.10. Antioxidative Capacity

The stable free radical 2,2-diphenyl-1-picrylhydrazyl (DPPH) was used to determine the antioxidant capacity of the produced VOO. The measurements were carried out using electron paramagnetic resonance spectroscopy (EPR) following the method described by Markić et al. [47] with some minor modifications. In brief, a DPPH˙ solution (0.15 mM) in ethyl acetate was prepared 24 h before the measurements and kept in the dark at 4 °C to ensure complete dissolution of the radical. A total of 20 µL of VOO was added to 980 µL of DPPH˙; this mixture was mixed well and kept in the dark at room temperature for 27 min. Then, the capillary was filled with the mixture, which was then inserted into a standard EPR tube. The EPR spectra were recorded at room temperature using the Magnettech MS—5000 X (Magnettech ESR5000 Bruker, Billerica, MA, USA) after exactly 30 min of contact between the oil and the radical solution. The settings for the spectrometry were magnetic field modulation frequency (100 kHz), central field (337 mT), sweep range (12 mT), sweep time (30 s), microwave power (10 mW), and modulation amplitude (0.2 mT). The scavenging effect of VOO on DPPH radicals was determined by the amplitude (A_30_) of the EPR signal. The antioxidant capacity (AC) of VOO was defined as the percentage of DPPH˙ reduction and calculated according to Equation (2):

(2) $$AC=\frac{A_{0}-A_{30}}{A_{0}}\times 100$$

where A_0_ is the signal amplitude of the blank sample (DPPH˙ solution containing 20 µL of ethyl acetate).

### 2.11. Statistical Analysis

The effects of FTT on the oil yield, quality, and chemical composition of Croatian VOOs were evaluated using a full factorial experimental design with two independent variables: (i) treatment temperature (15, 20, 25, 30, 35, and 40 °C) and (ii) olive variety (Istarska Bjelica, Levantinka, and Oblica). Three consecutive production batches of VOOs were carried out for each variety under each FTT condition. All resulting oil samples were analyzed in triplicate.

To assess the impact of variety on the measured parameters, a one-way analysis of variance (ANOVA) was conducted. Additionally, the effect of FTT temperature on each variety included in the study was also evaluated using one-way ANOVA. When a significant effect of the temperature was observed, Tukey’s post hoc test was applied to perform multiple comparisons and identify differences among the produced samples. To further explore the influence of specific components on oxidative stability and antioxidant capacity, partial least squares (PLS) regression analysis was performed. The PLS model for oxidative stability included fatty acid composition (sum of saturated fatty acids—SFAs, monounsaturated fatty acids—MUFAs, and polyunsaturated fatty acids—PUFAs) and bioactive compounds (*α*-tocopherol and phenolic compounds) as predictors. For the antioxidant capacity model, only bioactive compounds were used as a predictor. Variable Importance in Projection (VIP) scores were calculated to determine the most influential variables, and model performance was evaluated using the coefficient of determination (R^2^).

To identify optimal FTT temperatures for each olive variety, response surface methodology (RSM) was employed. Optimization aimed to maximize the oxidative stability index (OSI), antioxidant capacity (AC), and volatile compounds resulting from the lipoxygenase pathway (Σ LOX), while minimizing the levels of volatiles resulting from oxidation (ΣOX) and microbial activity (ΣMBA). For each response variable, a polynomial regression model was fitted and evaluated using ANOVA, lack-of-fit tests, and R^2^ values. Numerical optimization was performed using a desirability function approach, with individual goals and relative importance set for each response variable to reflect their contribution to overall oil quality. Optimal pretreatment temperatures were identified for each variety, and predicted response values at these optimal conditions were generated from the fitted models to illustrate the expected improvements in oil quality.

All statistical analyses were conducted at a significance level of *p* = 0.05. ANOVA, Tukey’s test, and PLS regression were carried out using XLSTAT 2023 (Lumivero, Denver, CO, USA), while RSM analyses were performed using Design-Expert 10 (Stat-Ease, Inc., Minneapolis, MN, USA).

## 3. Results and Discussion

Flash thermal treatment (FTT) is an emerging technological innovation in virgin olive oil processing, proposed primarily as a pre-malaxation strategy to enhance phenolic content and improve the volatile compound profile of the oil.

The present study focused on VOOs produced at laboratory scale under controlled conditions from three Croatian autochthonous olive varieties harvested at the stage of ripeness recommended as optimal by local VOO producers.

The temperature points from 25 to 40 °C of olive paste thermal treatment were selected based on prior research indicating their relevance for optimizing oil extraction under low ambient temperatures [13,24,25,48,49]. In contrast, rapid cooling to 15 °C and 20 °C was evaluated as a potential mitigation strategy for elevated paste temperatures observed during harvesting in warmer climates, a scenario increasingly associated with the effects of climate change [28,29,30].

In all treatments tested, the target paste temperature (27 °C) was reached within the malaxation time. The time required was between 7 and 29 min, depending on the initial temperature of the paste. In particular, approximately 23, 21 and 7 min were needed for initial temperature of 15 °C, 20 °C and 25 °C, respectively. For the treatments that started at paste temperature of 30 °C, 35 °C and 40 °C, it took approximately 15, 20 and 29 min, respectively. The observed thermal inertia during malaxation is highly dependent on the initial paste temperature and can be considered an integral part of the thermal pretreatment effect, highlighting the need for optimization of preheating and cooling in the industry.

Previous studies have demonstrated that FTT influences the activity of endogenous enzymes [13,30] which play a critical role in determining the final chemical composition and sensory quality of VOO. To assess the biochemical impact of these treatments, in our previous study we evaluated model systems of two key endogenous enzymes, lipoxygenase and β-glucosidase. The results indicated that higher temperatures used for thermal treatment increased the activity of both enzymes [50], supporting their potential applicability in an innovative approach to improve the current VOO production plant/process.

### 3.1. Basic Quality Parameters and Processing Yield

Thermal treatment of the olive paste prior to malaxation did not compromise the basic quality of any VOO produced at laboratory scale Abencor system. All oils remained within the “extra virgin olive oil” category (Table 1), as defined by current EU standards [51].

The acidity was significantly influenced by the health status of the olive fruit of Istarska Bjelica (*p* ≤ 0.01) and Oblica (*p* ≤ 0.001). However, the values of the free fatty acids were within a very narrow range of only 0.04 percentage units in Istarska Bjelica and 0.06 percentage units in Oblica, with few treatments being significantly different compared to the control. Other researchers investigating the effects of FTT on the quality of VOO have found no increase in acidity [27,30,52].

All thermal treatments prior to malaxation led to a statistically significant increase (between 47% and 81%) in the peroxide value (PV) compared to the untreated control. Acceleration of the oxidation process in our trial is most likely a consequence of the discontinuous phase of the FTT under the laboratory conditions used in this work. Despite this rise, PV levels in all samples remained well below the regulatory limit of 20 meq O_2_/kg, as stipulated by EU Regulations [51]. Similar results were reported by Fiori et al. [52] for VOO produced with FTT in industrial facilities, while Guerrini et al. [27], who produced VOO with FTT on a laboratory scale, together with others who used industrial facilities, did not achieve any PV increase [24,28,29]. However, the specific extinction coefficients at 232 nm (K_232_), which similarly to PV serves as indicator of the primary oxidation products in virgin oils, did not exhibit a significant increase across treatments. The reason may be that K_232_ includes only primary oxidation products formed from polyunsaturated fatty acids by photooxidation, while the peroxide number also includes those formed from oleic acid, which predominates in olive oil. The moderate oxidative deterioration of the oil under the applied conditions is also reflected by the fact that there was no statistically significant increase in K_268_ (except in Levantinka at 25 °C), which is associated with the presence of secondary oxidation products in virgin oils. Nevertheless, due to the potentially pro-oxidative effects of elevated temperatures as well as the influence of the discontinuous FTT process as a pretreatment for malaxation under laboratory conditions, additional indicators of oxidative stability were included in the study, which are discussed later in this document.

One of the biggest challenges in conventional VOO production is the relatively low oil yield: 10–20% of the oil remains trapped in the cell–matrix or in the colloidal system of the olive paste and part is bound in an emulsion with the vegetable water [53]. Recent research has focused on improving extraction efficiency through the use of innovative technologies, often in combination with malaxation. Notably, the use of pulsed electric fields (PEF) has shown promising results [54,55,56,57], as have and high hydrostatic pressure and ultrasound treatments [57,58]. Moreover, combining several of these innovative techniques has demonstrated additional potential to enhance olive oil extraction and quality [59]. In contrast, thermal treatment of olive paste, either applied alone or in combination with malaxation, has not consistently improved oil yield [24,25,28,60]. Our results are similar. Differences in olive yield within each variety, related to different temperature pretreatments, were not statistically significant compared to the control.

### 3.2. Volatile Compounds

Volatile compounds, both major and minor, play a critical role in determining the sensory quality and consumer perception of virgin olive oils (VOOs). Their composition is influenced by a multitude of factors, including olive cultivar, geographical origin, fruit ripeness, and technological parameters—particularly malaxation time and temperature.

In accordance with the classification proposed by Cecchi et al. [5], the volatile compounds identified in this study were grouped into three primary categories based on their biochemical, oxidative, or microbiological origins. Furthermore, compounds were classified into three levels of identification reliability, following the criteria established by Mikrou et al. [61]. Tables S3 and S4 (Supplementary Material) contain the results of the analysis of volatiles in samples of VOO prepared on a laboratory scale in Abencor using a discontinuous FTT process as a pretreatment for malaxation.

Table S3 presents the concentrations of volatile compounds generated via the lipoxygenase (LOX) pathway, which are largely responsible for the green and fruity sensory notes characteristic of high quality VOOs. In the control samples, clear varietal differences in the volatile profiles were observed. These findings align with previous studies on Croatian VOOs also produced at laboratory scale [35,62,63]. In comparison to control, flash thermal treatment significantly affected the sum of LOX-derived volatiles only in the Istarska Bjelica variety, where the concentration of total LOX volatiles achieved at 20 °C and 35 °C was significantly lower compared to control sample. Since statistical significance was not confirmed for the extreme temperature points (pretreatment of Istarska Bjelica at 15 °C and 40 °C), it can be assumed that disadvantages of laboratory-scale process (such as the exposure of the olive paste to oxygen and the additional time required to reach the malaxation temperature) could be the cause of such inconsistencies. Regardless, the results still highlight the complex, genotype-dependent response of the LOX pathway to thermal pretreatment, as also reported by other authors [10,30,64,65,66].

Beyond the LOX pathway, volatile compounds in VOOs can also arise from autoxidation and/or photooxidation processes. In the present study, three oxidation-derived volatile compounds were identified (Table S4 in the Supplementary Material). While the accumulation of nonanal was relatively uniform in all three varieties, a decrease in 2,4-hexadienal was observed in Istarska Bjelica compared to the other two varieties. This lower level reflects the higher oxidation resistance of Istarska Bjelica, as indicated by the induction time (Chapter 3.6). A statistically significant decrease in 2,4-hexadienal and consequently in the sum of volatile oxidation products was observed in the VOO of Oblica when pretreated at 40 °C.

In addition to oxidative processes, microbiological activity during post-harvest olive storage can substantially contribute to volatile formation. The native microbiota present on olive fruit surfaces, including bacteria, yeasts, and molds, can proliferate during storage and metabolize fruit constituents, thereby producing various fermentation-related volatiles [5]. In this study, two microbial metabolites, 3-methylbutanal and 2-methylbutanal, were identified and quantified. Statistically significantly higher concentrations compared to control oils were observed at a pretreatment temperature of 25 °C in oils derived from Istarska Bjelica and Oblica, while Levantinka showed no significant changes at all. Given the short duration of the treatment, the lack of similarity between varieties, and the lack of a clear trend depending on the pretreatment temperature, the observed changes could not be associated with microbial activity during sample preparation.

Although FTT appeared to reduce the overall concentration of volatile compounds associated with sensory defects across all three cultivars, the potential occurrence of undesirable sensory attributes, such as rancid or fusty/muddy sediment notes, in VOOs produced with FTT cannot be entirely excluded.

### 3.3. Phenolic Compounds

Phenolic compounds in VOO have been extensively studied due to their well-established health-promoting properties [1,67,68]. These bioactive molecules exhibit potent antioxidant activity, primarily attributed to their unique chemical structures. In olives, phenolics are biosynthesized through complex chemical and enzymatic pathways. To date, more than 30 distinct phenolic compounds have been identified in VOO, with total phenolic content (TPC) ranging from 40 to more than 4000 mg/kg [69]. The concentration and composition of phenolics in olives are modulated by various factors, including variety, ripening stage, climatic conditions, as well as agronomic and technological practices. Table 2 presents the phenolic profiles of oils extracted using a laboratory-scale Abencor system with and without the application of FTT.

The data underscore the significant influence of variety on all phenol components. Notably, the control sample of Istarska Bjelica variety exhibited an exceptionally high TPC of 773 mg/kg, approximately 2.4 times higher than that of Levantinka (328 mg/kg) and 1.8 times higher than that of Oblica (429 mg/kg). In Istarska Bjelica oil, ligstroside aglycones and oleocanthal were the predominant phenolic compounds. Conversely, Levantinka oil contained the highest concentrations of oleacein and oleocanthal, whereas Oblica oil was characterized by a predominance of oleuropein and ligstroside aglycones. The TPCs recorded for Istarska Bjelica slightly exceeded values reported previously [35,70,71] (414–685 mg/kg), while the TPCs for Levantinka and Oblica were within the range reported for these varieties (212–694 mg/kg and 50–791 mg/kg, respectively) [35,63,72].

Probably due to the different activity of endogenous enzymes determined by the genetic characteristics of the variety [13,28,29], the influence of FTT (temperatures of 25 °C and above) was significant only for the VOOs of the Oblica variety. Heating led to increased concentrations of oleacein and oleocanthal, accompanied by a decrease in tyrosol, ligstroside aglycones and *p*-coumaric acid. These findings are consistent with previous reports indicating that elevated malaxation temperatures can enhance the concentrations of oleacein and oleocanthal in VOOs produced both at laboratory scale [71,73,74] and industrial plant [13]. Several studies [48,75,76] have attributed such increases to the thermal activation of endogenous enzymes that facilitate the formation of phenolic compounds during malaxation or promote their release by disrupting cellular structures. However, the potential adverse effects of elevated olive paste temperatures, as we noticed on Oblica ligstroside aglycones, *p*-coumaric acid and partially tyrosol, must also be considered. Numerous studies have reported phenolic degradation via enzymatic oxidation mediated by polyphenol oxidase (PPO) and peroxidase (POD) produced both on laboratory scale [77,78] and industrial plant [79]. In addition to heating, cooling the olive paste after crushing has also been shown to have a positive effect on the concentration and profile of phenolic compounds [28,29,30], but this was not the case in our study. Ultimately, the phenolic composition of VOO reflects a complex interplay among enzymatic activity, thermal stability of key enzymes (e.g., β-glucosidase, esterase, PPO, POD), and the partitioning behavior of phenolics between the oil and aqueous phases. All these processes are strongly influenced by the temperature of the olive paste during processing [13,14,26,29,30,31,80].

### 3.4. Tocopherols

Tocopherols are lipid-soluble compounds synthesized exclusively by plants, and they play a crucial role in protecting biological membranes and oils from oxidative degradation. In VOO, *α*-tocopherol is the predominant isoform, typically representing more than 95% of the total tocopherol content. The concentration of tocopherols in olive fruits appears to be primarily determined by genetic and agronomic factors, rather than by technological or processing conditions.

Table 3 presents the concentrations of the tocopherol isoforms detected in the VOO samples extracted using a laboratory-scale Abencor system with and without the application of FTT. The control oils illustrate clear varietal differences: Istarska Bjelica exhibited markedly lower *α*-tocopherol concentrations compared to Levantinka and Oblica. Previous studies have reported *α*-tocopherol levels in Istarska Bjelica ranging from 89 to 96 mg/kg [81,82,83]. In the current study, *α*-tocopherol levels in Istarska Bjelica were even lower than this published range. Additionally, *γ*-tocopherol was not detected in this variety. The Levantinka control sample contained 273 mg/kg of *α*-tocopherol, consistent with previously reported values [84]. Similarly, the tocopherol concentration in the Oblica control sample aligns with data published by Šarolić et al. [84] and Jukić Špika et al. [85].

Each olive variety exhibited a distinct response to FTT across the range of applied temperatures. In Istarska Bjelica, no statistically significant differences in tocopherol content were observed between the control and treated samples, regardless of whether the pretreatment involved cooling or heating. The tocopherols appeared to remain stable under thermal stress, likely due to the synergistic action of oleacein, which has been shown to effectively regenerate *α*-tocopherol by reducing the *α*-tocopheroxyl radical [86]. All Istarska Bjelica oil samples, irrespective of FTT temperature, contained high concentrations of oleacein (Table 2), which may contribute to the preservation of tocopherols. In contrast, the Levantinka variety, which also contains significant concentrations of oleacein, showed a statistically significant decrease in *α*-tocopherol content after cooling to 15 °C and 20 °C and after heating to 35 °C and 40 °C (the observed decreases were in the range from 13 to 15%). Considering that the oils of Istarska Bjelica contained 2.4 times more total phenols than that of Levantinka, it is very likely that not only oleacein, but also other phenolic substances in the olive paste protected tocopherols from oxidation during malaxation.

In contrast to Levantinka, cooling pretreatment did not cause a reduction in *α*-tocopherol in Oblica oils, while decline under heating pretreatment at 35 °C and 40 °C compared to control was stronger than in Levantinka samples (it ranged from 23% to 24%). The observed decline in tocopherol content is likely attributable to its role as an antioxidant, during which it is consumed in neutralizing oxidative radicals. This mechanism has been described by Rastrelli et al. [87], who reported the degradation of *α*-tocopherol under oxidative stress conditions. Supporting this, the Oblica oils subjected to FTT heating also showed the highest increase in primary oxidation products, as reflected by the peroxide value (PV, Table 1), and exhibited the shortest oxidative stability (induction period, Table 4), suggesting a correlation between tocopherol depletion and increased lipid oxidation. The protective role of phenolics in relation to *α*-tocopherol is not evident in case of Oblica, since the concentration of oleacein increased significantly at 35 °C and 40 °C while total phenols remained unchanged.

### 3.5. Fatty Acid Composition

The biosynthesis of fatty acids from malonyl-coenzyme A takes place in the plastid stroma of the olive fruit and is catalyzed by a series of specific enzymes through multiple iterative cycles. These fatty acids are subsequently esterified into triacylglycerols via the Kennedy pathway (also known as the glycerol-3-phosphate pathway) [88,89].

The fatty acid composition of virgin olive oil is influenced by several factors, including latitude, climate, geographical origin, variety and ripeness of the fruit [90,91]. In our experiment, which was conducted using a laboratory-scale Abencor system (Table S5 in Supplementary Material), variety had a significant effect on all identified fatty acids (except eicosenoic acid). No significant deviations were found in the fatty acid profiles of the control samples compared to previously published data for oils from the same olive varieties also produced with the Abencor system [72,92,93,94].

When discontinuous flash thermal treatment (FTT) was applied, a slight but statistically significant increase in the percentage share of oleic acid and total MUFA in VOOs from Istarska Bjelica, palmitic acid in samples from Levantinka and Oblica, palmitoleic and linoleic acid together with total SFA and PUFA in Levantinka variety was observed. The differences in the mentioned fatty acid composition are too small to justify an effect of flash treatment.

The fatty acid composition of all VOO samples, both untreated and thermally pretreated, remained within the regulatory limits for extra virgin olive oil (EVOO) as defined by the applicable EU Regulation [51].

### 3.6. Oxidative Stability and Antioxidant Capacity

The oxidative stability of VOO refers to its resistance to oxidative degradation. A key parameter used to characterize this resistance is the oxidative stability index (OSI), defined as the induction period (IP)—the time required for a significant increase in the oxidation rate under accelerated conditions [95,96]. The results of the analysis of the VOOs produced with the Abencor laboratory system are shown in Table 4.

Antioxidant capacity is defined as the total amount of oxidants neutralized by antioxidant mechanisms, representing the cumulative antioxidant activity present in VOO [97]. Phenolic compounds are among the most potent antioxidants in VOO which act as chain-breaking antioxidants. These compounds react with lipid radicals to form stable, non-reactive radicals, thereby interrupting the propagation phase of lipid peroxidation. In VOO, phenolic compounds exhibit their antioxidant activity primarily by scavenging peroxyl and alkyl radicals, as well as by chelating trace amounts of transition metal ions that may catalyze oxidative reactions [98,99].

**Table 4:** Induction period (IP) and antioxidant capacity (AC) of controls and oils produced with flash thermal treatment.

Sample	IP (min) *	AC (% DPPH˙ Reduction) *
Istarska Bjelica	*p* ≤ 0.01	*p* = 0.379
Control	189 ± 8 b	60.07 ± 0.25
15 °C	206 ± 8 ab	58.77 ± 0.76
20 °C	197 ± 6 b	61.58 ± 2.98
25 °C	200 ± 9 ab	59.87 ± 1.01
30 °C	210 ± 5 ab	60.73 ± 1.54
35 °C	199 ± 7 ab	59.88 ± 1.10
40 °C	219 ± 5 a	59.53 ± 0.59
Levantinka	*p* = 0.253	*p* ≤ 0.001
Control	168 ± 11	50.18 ± 1.33 ab
15 °C	149 ± 4	50.00 ± 0.46 ab
20 °C	156 ± 10	51.77 ± 1.20 ab
25 °C	155 ± 9	44.53 ± 0.38 c
30 °C	170 ± 27	51.12 ± 1.46 ab
35 °C	145 ± 5	48.87 ± 1.53 b
40 °C	157 ± 7	52.53 ± 0.67 a
Oblica	*p* ≤ 0.001	*p* ≤ 0.001
Control	122 ± 6 a	65.70 ± 1.91 a
15 °C	119 ± 5 a	57.73 ± 1.55 b
20 °C	117 ± 2 a	63.20 ± 1.18 a
25 °C	103 ± 9 b	55.70 ± 2.13 b
30 °C	94 ± 1 bc	54.07 ± 0.70 bc
35 °C	88 ± 0 c	50.53 ± 0.81 cd
40 °C	88 ± 3 c	47.07 ± 1.10 d

* Variety had a significant effect (*p* ≤ 0.05); presented *p*-values indicate the effect of temperature within each variety. Where significant (*p* ≤ 0.05), Tukey’s multiple comparison test was conducted and different letters within column subsections indicate significant differences. All values are mean ± standard deviation of three consecutive production batches.

Tocopherols, another important class of antioxidants present in VOO, also scavenge peroxyl radicals. In addition, they are among the most effective natural quenchers of singlet oxygen, converting it to its ground state and thereby inhibiting singlet oxygen-mediated oxidation of unsaturated fatty acids [95]. The evaluation of the antioxidant capacity of EVOO reflects not only the contributions of phenolic compounds and tocopherols, but also of other minor antioxidants, which together enhance the nutritional value and oxidative stability of the oil through synergistic interactions. In this study, the antioxidant capacity of VOO obtained from olive paste subjected to FTT was assessed using the stable free radical DPPH˙. The results are presented in Table 4 as the percentage reduction of the DPPH˙ radical.

Given the differences in phenolic content, tocopherol composition, and fatty acid profiles among the studied olive varieties, it was anticipated that each would exhibit a distinct response to FTT in terms of oxidative stability and antioxidant capacity [3].

The Istarska Bjelica variety demonstrated strong tolerance to FTT, as evidenced by an increase in IP during both the cooling and heating phases, although a statistically significant increase in IP compared to the control was observed only after heating to 40 °C. This stability can be attributed to its robust antioxidant capacity, which remained unaffected by thermal treatment. Notably, Istarska Bjelica possesses the highest concentration of phenolic compounds among the tested varieties (Table 2), particularly oleocanthal, oleuropein aglycone, and hydroxytyrosol—compounds recognized for their potent antioxidant activity [3]. In the Levantinka variety, a downward trend in IP was observed although not statistically significant. Antioxidant capacity also did not differ significantly from the control, except at 25 °C. The most pronounced decline in oxidative stability, especially under heating conditions, was observed in oils derived from the Oblica variety. This variety is characterized by a high content of PUFAs, particularly C18:2, which is nearly twice that of the other tested varieties. Notably, the PUFA levels were not significantly affected by the FTT treatment (Table S5). However, heating during FTT treatment led to a significant reduction in tyrosol and ligstroside aglycone levels (Table 2), as well as *α*-tocopherol content (Table 3). The thermally induced decrease in antioxidants, particularly *α*-tocopherol which plays a key role in protecting C18:2 against oxidation [100], resulted in a significant reduction in the induction period. A reduction in IP, linked to the loss of phenolic compounds after rapid preheating of olive paste at 38 °C, was also observed by Fiori et al. [52]. Although Oblica exhibited higher antioxidant capacity than Levantinka, applying FTT at all temperatures except 20 °C caused a statistically significant decline in antioxidant activity.

Given the observed influence of PUFAs, phenolic compounds and α-tocopherol on the induction period and antioxidant capacity of the Oblica variety, we aimed to evaluate the extent to which these and other bioactive components in VOO from the three tested varieties, subjected to FTT, contribute to overall oxidative stability and antioxidant potential.

To this end, partial least squares (PLS) regression analysis was applied to investigate the influence of specific oil constituents on oxidative stability and antioxidant capacity. For the oxidative stability model (measured by the induction period, IP), predictor variables included the fatty acid composition and the quantified bioactive compounds. The resulting model is presented in Equation (3):

IP = −31.515 + 2.716 × SFA + 1.430 ∗ MUFA − 1.992 ∗ PUFA − 0.056 ∗ *α*-TOCO + 0.4801 ∗ HTY − 0.115 ∗ OLEA + 0.39 ∗ OLEU AGL + 0.929 ∗ TY + 0.135 ∗ OLEC + 0.681 ∗ MH OLEC + 0.096 ∗ LIG AGL − 0.652 ∗ *p*-CA + 0.023 ∗ TPC (3)

The abbreviations in the equation stand for: SFA—sum of saturated fatty acids, MUFA—sum of monounsaturated fatty acids, PUFA—sum of polyunsaturated fatty acids, *α*-TOCO—*α*-tocopherol, HTY—hydroxytyrosol, TY—tyrosol, OLEA—oleacein, OLEC—oleocanthal, OLEU AGL—sum of oleuropein aglycones, LIG AGL—sum of ligstroside aglycones, MH OLEC—methyl hemiacetal of oleocanthal, *p*-CA—*p*-coumaric acid, TPC—total phenols.

**Figure 1:**
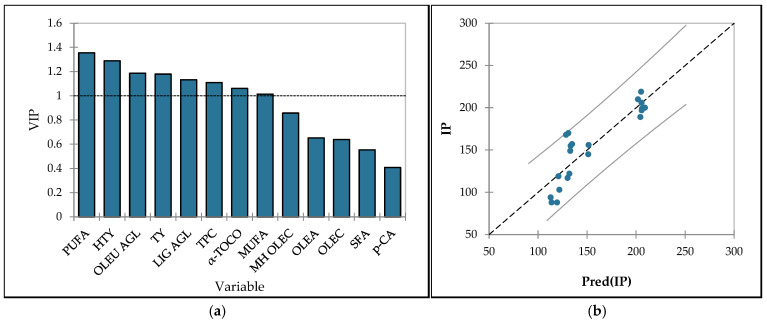
(**a**) Variable importance in the projection (VIP) of induction period—IP (min) with the variables SFA—sum of saturated fatty acids, MUFA—sum of monounsaturated fatty acids, PUFA—sum of polyunsaturated fatty acids, *α*-TOCO—*α*-tocopherol, HTY—hydroxytyrosol, TY—tyrosol, OLEA—oleacein, OLEC—oleocanthal, OLEU AGL—sum of oleuropein aglycones, LIG AGL—sum of ligstroside aglycones, MH OLEC—methyl hemiacetal of oleocanthal, *p*-CA—*p*-coumaric acid, TPC—total phenols. The dashed black line represents the significance threshold for VIP score. (**b**) The predicted versus actual values of induction period. The gray solid line represents the 95% confidence interval.

The model demonstrated strong predictive power, with a coefficient of determination (R^2^) of 0.788 and a cross-validated R^2^ (Q^2^) of 0.733, indicating that approximately 78.8% of the variability in oxidative stability could be explained by the included predictors. Variable Importance in Projection (VIP) scores (Figure 1a) identified key contributors, with PUFA, hydroxytyrosol, oleuropein aglycones, tyrosol, ligstroside aglycones, TPC, *α*-tocopherol, and MUFA all exhibiting VIP > 1.0. The predicted vs. observed plot (Figure 1b) confirmed the model’s robustness, with most data points aligning closely with the 1:1 reference line. Model predictions indicate that oxidative stability is positively influenced by the presence of MUFA, as well as hydroxytyrosol, tyrosol, oleuropein and ligstroside aglycones, and total phenolic content. Conversely, the IP is negatively affected by elevated concentrations of PUFAs, due to their high susceptibility to oxidation, as well as by *α*-tocopherol and oleacein. Similarly, Serrano et al. [101] reported a strong positive effect of C18:1 and oleuropein aglycones on IP, while C18:2 and oleacein exhibited a negative impact.

For antioxidant capacity (AC), only bioactive compounds (α-tocopherol and phenolics) were considered. The corresponding PLS model is shown in Equation (4):

AC = 46.739 – 0.007 ∗ *α*-TOCO + 0.053 ∗ HTY − 0.015 ∗ OLEA + 0.005 ∗ OLEA AGL + 0.1004 ∗ TY + 0.001 ∗ OLEC + 0.118 ∗ MH OLEC + 0.016 ∗ LIG AGL + 0.250 ∗ *p*-CA + 0.003 ∗ TPC (4)

With a coefficient of determination R^2^ = 0.583. The abbreviations in the equation stand for *α*-TOCO—*α*-tocopherol, HTY—hydroxytyrosol, TY—tyrosol, OLEA—oleacein, OLEC—oleocanthal, OLEU AGL—sum of oleuropein aglycones, LIG AGL—sum of ligstroside aglycones, MH OLEC—methyl hemiacetal of oleocanthal, *p*-CA—*p*-coumaric acid, TPC—total phenols.

**Figure 2:**
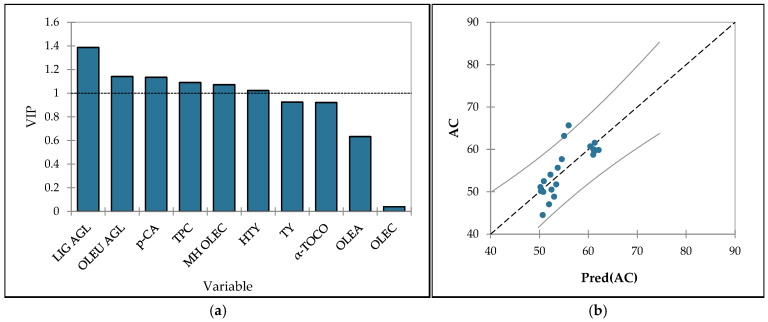
(**a**) Variable importance in the projection (VIP) of antioxidant capacity—AC (% red. DPPH˙) with the variables *α*-TOCO—*α*-tocopherol, HTY—hydroxytyrosol, TY—tyrosol, OLEA—oleacein, OLEC—oleocanthal, OLEU AGL—sum of oleuropein aglycones, LIG AGL—sum of ligstroside aglycones, MH OLEC—methyl hemiacetal of oleocanthal, *p*-CA—*p*-coumaric acid, TPC—total phenols. The dashed black line represents the significance threshold for VIP score. (**b**) The predicted versus actual values of antioxidant capacity. The gray solid line represents the 95% confidence interval.

The model for antioxidant capacity yielded an R^2^ of 0.583 and a Q^2^ of 0.515, indicating moderate predictive strength. VIP analysis (Figure 2a) highlighted ligstroside aglycons, oleuropein aglycons, *p*-coumaric acid, TPC, methyl hemiacetal of oleocanthal, and hydroxytyrosol (VIP > 1) as the main contributors to AC. Compounds such as tyrosol, *α*-tocopherol, oleacein, and oleocanthal showed VIP scores < 1, suggesting a lesser impact under the conditions studied. Interestingly, similar to the case of IP, both *α*-tocopherol and oleacein demonstrated a negative impact on antioxidant capacity. In contrast, other phenolic compounds showed a positive effect. Previous studies have reported that hydroxytyrosol and oleuropein derivatives and ligstroside aglycones possess the strongest antioxidant activity among the phenolic compounds found in VOO [102,103]. The predicted vs. actual values plot (Figure 2b) further supported the model’s validity. However, some scatter was evident, particularly at higher antioxidant capacity values, indicating potential limitations in the model’s accuracy across the full range of observations. These findings are consistent with the established roles of major phenolic compounds and tocopherols as potent antioxidants in olive oil, although the moderate R^2^ indicates that additional factors may also contribute significantly.

### 3.7. Optimization

Response surface methodology (RSM) was used to individually optimize FTT temperatures for each of the three Croatian olive varieties used in this study. The aim was to enhance the oxidative stability index (OSI), indicated by induction period (IP), antioxidant capacity (AC) and volatiles derived from the lipoxygenase pathway (Σ LOX), while minimizing oxidation-derived volatiles (Σ OX) and those associated with microbial activity (Σ MBA). Polynomial models were developed for each parameter and tested for suitability using ANOVA, coefficients of determination (R^2^), and lack-of-fit tests (Table 5).

The best-fitting models were obtained for the Oblica variety. All models showed high statistical significance (*p* < 0.05), with R^2^ values ranging from 0.659 to 0.908, indicating they explained a substantial portion of the observed variability. Additionally, non-significant lack-of-fit results (*p* > 0.05) for IP, Σ LOX and Σ OX, confirming the suitability of the models to capture the experimental data. Second-order polynomial models were also developed for the remaining two varieties. However, the ANOVA results showed statistical significance only for the IP model for Istarska Bjelica and the Σ MBA model for Levantinka.

For the Istarska Bjelica variety, the cooling of the olives to 18.9 °C was predicted to increase the IP value by 6.4% and the AC value by 1.2%. Although a decrease in Σ LOX volatiles of about 15% was expected, Σ OX was expected to decrease by 4% and Σ MBA by 42%. In contrast, for the Oblica variety, a decrease in OSI and AC (2% and 10%, respectively) was predicted, but an improvement in sensory properties due to a 15% increase in Σ LOX volatile components and a 75% decrease in Σ MBA. For the Levantinka variety, an improvement in AC was predicted by applying the optimal FTT temperature, although a reduction in both IP and Σ LOX volatiles was also expected.

## 4. Conclusions

The objective of this study was to evaluate the impact of flash thermal pretreatment of olive paste prior to malaxation on the oxidative stability and antioxidant capacity of VOO derived from three Croatian indigenous olive varieties. Each variety exhibited a distinct response to FTT, applied through both heating and cooling, which manifested in changes in the chemical profile of the resulting VOO.

Among the tested varieties, Istarska Bjelica demonstrated the highest resistance to oxidative degradation. OSI increased under both heating and cooling conditions relative to the untreated control, with a statistically significant enhancement at 40 °C corresponding to a 16% increase in induction time. The antioxidant capacity of this variety remained unaffected by all thermal treatments. The improvement in IP and robustness of AC is primarily attributed to the highest concentrations of phenolic compounds, which were identified as the most important factors for OSI and AC via PLS regression analysis.

In contrast, VOO obtained from the Oblica variety was most susceptible to oxidative deterioration. FTT via heating resulted in a pronounced reduction in OSI (23–28%) and a decline in AC (12–28%) across all treatment temperatures, except 20 °C. *α*-tocopherol and *γ*-tocopherol levels were significantly diminished under both heating and cooling conditions. Heating treatments notably reduced the total content of ligstroside aglycones (by 30–68%) and *p*-coumaric acid (by 16–25%), phenolic compounds relevant to OSI and AC. This variety also exhibited the lowest MUFA (~70%) and highest PUFA (~12%) levels among the three. However, thermal pretreatment at 30, 35, and 40 °C led to a marked increase in the concentrations of olacein (1.9–2.8 times relative to control) and oleocanthal (1.6–2 times).

Levantinka exhibited intermediate sensitivity. A decrease in OSI and AC was also observed after FTT, but the Levantinka has a longer IP than the Oblica, and the decrease in AC (11%) was only pronounced at 25 °C. Although it has the lowest concentration of phenolic compounds compared to the other two varieties, Lavantinka is characterized by the highest MUFA and the lowest PUFA levels. The *α*-tocopherol content, which is the highest compared to other two varieties, decreased significantly both when cooled and when heated to 40 °C.

The optimal FTT conditions for maximizing VOO oxidative stability, antioxidant capacity, and favorable volatile profiles predicted using response surface methodology (RSM) are as follows: 18.9 °C for Istarska Bjelica, 15.4 °C for Levantinka, and 15.5 °C for Oblica.

## Figures and Tables

**Table 1:** Basic quality parameters (acidity, peroxide value—PV and K values) and extraction yield of controls and oils produced with flash thermal treatment.

Sample	Acidity (% Oleic Fatty Acid) *	PV (Meq O_2_/kg)	K Values			Yield (%) *
			K_232_ *	K_268_ *	ΔK *	
Istarska Bjelica	*p* ≤ 0.01	*p* ≤ 0.001	*p* ≤ 0.05	*p* = 0.522	*p* ≤ 0.001	*p* ≤ 0.05
Control	0.42 ± 0.03 a	2.9 ± 0.5 d	1.92 ± 0.12 a	0.23 ± 0.03	0.00 ± 0.00 a	24.70 ± 1.02 a
15 °C	0.38 ± 0.01 b	4.8 ± 0.1 abc	1.89 ± 0.06 a	0.19 ± 0.02	−0.01 ± 0.01 b	25.18 ± 0.59 a
20 °C	0.39 ± 0.01 ab	5.3 ± 0.1 ab	2.02 ± 0.01 a	0.23 ± 0.02	−0.01 ± 0.00 b	25.72 ± 0.14 a
25 °C	0.39 ± 0.00 ab	6.0 ± 0.3 a	2.00 ± 0.04 a	0.21 ± 0.01	−0.01 ± 0.00 b	26.69 ± 0.58 a
30 °C	0.39 ± 0.01 ab	4.5 ± 1.0 bc	2.06 ± 0.13 a	0.23 ± 0.08	−0.01 ± 0.00 b	25.86 ± 0.30 a
35 °C	0.42 ± 0.01 ab	3.4 ± 0.9 cd	2.10 ± 0.07 a	0.25 ± 0.03	−0.01 ± 0.00 b	25.98 ± 0.39 a
40 °C	0.42 ± 0.01 a	3.4 ± 0.1 cd	2.07 ± 0.07 a	0.20 ± 0.04	−0.01 ± 0.00 b	25.69 ± 0.27 a
Levantinka	*p* = 0.071	*p* ≤ 0.05	*p* = 0.525	*p* ≤ 0.05	*p* ≤ 0.001	*p* = 0.127
Control	0.24 ± 0.01	3.3 ± 0.2 b	1.7 ± 0.05	0.10 ± 0.01 b	−0.01 ± 0.01 a	12.22 ± 0.17
15 °C	0.24 ± 0.01	6.9 ± 0.8 a	1.67 ± 0.09	0.15 ± 0.05 ab	0.00 ± 0.00 a	12.44 ± 0.24
20 °C	0.25 ± 0.01	4.9 ± 1.0 ab	1.62 ± 0.04	0.12 ± 0.01 ab	0.00 ± 0.00 a	12.95 ± 0.48
25 °C	0.22 ± 0.00	3.9 ± 1.6 ab	1.69 ± 0.06	0.16 ± 0.01 a	0.00 ± 0.00 a	12.22 ± 0.58
30 °C	0.23 ± 0.02	4.6 ± 1.6 ab	1.69 ± 0.09	0.13 ± 0.01 ab	0.00 ± 0.00 a	12.36 ± 0.27
35 °C	0.23 ± 0.01	5.3 ± 1.5 ab	1.66 ± 0.02	0.11 ± 0.01 ab	−0.01 ± 0.00 a	12.43 ± 0.57
40 °C	0.24 ± 0.00	6.3 ± 0.6 a	1.71 ± 0.03	0.12 ± 0.01 ab	−0.01 ± 0.00 a	13.03 ± 0.32
Oblica	*p* ≤ 0.001	*p* ≤ 0.001	*p* = 0.546	*p* = 0.470	*p* ≤ 0.001	*p* ≤ 0.001
Control	0.30 ± 0.01 bc	3.5 ± 0.2 d	1.98 ± 0.08	0.20 ± 0.05	−0.01 ± 0.00 b	13.58 ± 0.32 ab
15 °C	0.30 ± 0.01 c	4.5 ± 0.4 bcd	1.93 ± 0.10	0.22 ± 0.07	0.00 ± 0.01 a	12.81 ± 0.39 b
20 °C	0.30 ± 0.00 bc	4.3 ± 0.7 cd	1.88 ± 0.06	0.19 ± 0.05	0.00 ± 0.00 a	12.89 ± 0.36 b
25 °C	0.27 ± 0.01 d	4.3 ± 0.7 cd	1.80 ± 0.11	0.17 ± 0.04	0.00 ± 0.00 a	12.71 ± 0.23 b
30 °C	0.32 ± 0.00 ab	6.5 ± 0.3 abc	2.10 ± 0.06	0.19 ± 0.02	−0.01 ± 0.00 b	13.67 ± 0.39 ab
35 °C	0.33 ± 0.01 a	6.8 ± 1.4 ab	1.45 ± 1.08	0.12 ± 0.09	−0.01 ± 0.00 b	13.93 ± 0.15 a
40 °C	0.32 ± 0.01 abc	7.7 ± 1.3 a	2.09 ± 0.02	0.19 ± 0.02	−0.01 ± 0.00 b	13.94 ± 0.21 a

* Variety had a significant effect (*p* ≤ 0.05); presented *p*-values indicate the effect of temperature within each variety. Where significant (*p* ≤ 0.05), Tukey’s multiple comparison test was conducted and different letters within column subsections indicate significant differences. All values are mean ± standard deviation of three consecutive production batches.

**Table 2:** Composition of phenolic compounds (expressed as tyrosol) of controls and oils produced with flash thermal treatment.

Sample	Phenolic Compound (mg/kg)								
	Hydroxytyrosol *	Tyrosol *	Oleacein *	Oleocanthal *	Methyl Hemiacetal of Oleocanthal *	Σ of Oleuropein Aglycones *^§^	Σ of Ligstroside Aglycones *^£^	*p*-Coummaric Acid *	Total Phenolic *
Istarska Bjelica	*p* = 0.324	*p* = 0.368	*p* = 0.371	*p* = 0.538	*p* ≤ 0.05	*p* = 0.231	*p* = 0.234	*p* ≤ 0.05	*p* = 0.845
Control	36 ± 4	16 ± 1	77 ± 12	106 ± 8	32 ± 4 a	330 ± 42	169 ± 16	7 ± 0 b	773 ± 85
15 °C	39 ± 2	18 ± 1	70 ± 4	107 ± 2	24 ± 1 ab	368 ± 18	163 ± 3	8 ± 1 ab	798 ± 25
20 °C	41 ± 3	20 ± 2	57 ± 2	102 ± 2	21 ± 1 b	360 ± 16	168 ± 6	9 ± 1 ab	778 ± 21
25 °C	42 ± 5	21 ± 7	67 ± 24	112 ± 11	29 ± 7 ab	327 ± 20	156 ± 1	10 ± 0 a	765 ± 37
30 °C	38 ± 2	18 ± 1	79 ± 15	111 ± 8	23 ± 3 ab	344 ± 25	157 ± 7	8 ± 1 ab	775 ± 50
35 °C	40 ± 3	19 ± 1	71 ± 4	108 ± 2	24 ± 1 ab	360 ± 23	167 ± 5	9 ± 1 ab	798 ± 24
40 °C	40 ± 1	18 ± 1	76 ± 2	111 ± 1	24 ± 0 ab	366 ± 9	165 ± 3	9 ± 0 ab	809 ± 10
Levantinka	*p* = 0.791	*p* = 0.701	*p* = 0.421	*p* = 0.383	*p* = 0.603	*p* ≤ 0.05	*p* ≤ 0.05	*p* = 0.102	*p* ≤ 0.01
Control	15 ± 1	5 ± 0	83 ± 8	83 ± 4	15 ± 2	69 ± 6 ab	54 ± 5 ab	5 ± 0	328 ± 20 ab
15 °C	16 ± 2	9 ± 4	60 ± 10	79 ± 3	11 ± 10	62 ± 12 ab	57 ± 12 ab	5 ± 1	299 ± 41 ab
20 °C	24 ± 14	12 ± 10	60 ± 26	82 ± 13	23 ± 9	83 ± 11 a	69 ± 3 a	5 ± 1	356 ± 10 a
25 °C	16 ± 13	10 ± 9	63 ± 27	72 ± 17	17 ± 11	52 ± 5 b	38 ± 13 b	4 ± 1	273 ± 27 b
30 °C	14 ± 1	5 ± 0	81 ± 4	85 ± 1	15 ± 1	70 ± 12 ab	58 ± 3 ab	4 ± 0	333 ± 20 ab
35 °C	27 ± 25	14 ± 4	54 ± 37	74 ± 17	22 ± 15	67 ± 10 ab	57 ± 4 ab	4 ± 0	318 ± 19 ab
40 °C	17 ± 1	7 ± 1	82 ± 10	91 ± 2	13 ± 3	73 ± 7 ab	63 ± 8 a	5 ± 0	351 ± 18 ab
Oblica	*p* ≤ 0.05	*p* ≤ 0.001	*p* ≤ 0.001	*p* ≤ 0.001	*p* = 0.059	*p* = 0.423	*p* ≤ 0.001	*p* ≤ 0.001	*p* = 0.146
Control	23 ± 2 a	12 ± 2 a	56 ± 12 b	61 ± 5 c	21 ± 5	138 ± 22	106 ± 10 a	12 ± 0 a	429 ± 50
15 °C	19 ± 1 a	8 ± 1 abc	57 ± 11 b	63 ± 6 bc	20 ± 1	123 ± 10	96 ± 3 ab	12 ± 0 a	397 ± 2
20 °C	21 ± 1 a	9 ± 0 abc	62 ± 4 b	62 ± 3 c	22 ± 1	115 ± 5	103 ± 2 a	10 ± 1 ab	405 ± 6
25 °C	17 ± 2 a	8 ± 1 bc	65 ± 3 b	70 ± 1 b	17 ± 3	129 ± 19	80 ± 8 ab	10 ± 1 ab	395 ± 25
30 °C	10 ± 5 a	6 ± 1 c	114 ± 15 a	97 ± 9 a	18 ± 2	103 ± 4	74 ± 11 b	9 ± 0 b	430 ± 13
35 °C	10 ± 5 a	7 ± 1 c	106 ± 19 ab	96 ± 13 ab	16 ± 2	90 ± 7	71 ± 6 b	10 ± 0 b	405 ± 25
40 °C	21 ± 11 a	10 ± 3 ab	154 ± 14 a	132 ± 12 a	14 ± 5	119 ± 64	33 ± 20 c	9 ± 1 b	496 ± 99

* Variety had a significant effect (*p* ≤ 0.05); ^§^ sum of oleuropein aglycone isomers, and mono- and dialdehydic form of oleuropein aglycone; ^£^ sum of ligstroside aglycone, mono- and dialdehydic form of ligstroside aglycone, and oleokoronal; presented *p*-values indicate the effect of temperature within each variety. Where significant (*p* ≤ 0.05), Tukey’s multiple comparison test was conducted and different letters within column subsections indicate significant differences. All values are mean ± standard deviation of three consecutive production batches.

**Table 3:** Tocopherol content expressed as *α*-tocopherol of controls and oils produced with flash thermal treatment.

Sample	Tocopherols (mg/kg)	
	*α*-Tocopherol *	*γ*-Tocopherol *
Istarska Bjelica	*p* ≤ 0.05	**
Control	75 ± 4 a	nd
15 °C	73 ± 6 a	nd
20 °C	83 ± 0 a	nd
25 °C	78 ± 4 a	nd
30 °C	76 ± 6 a	nd
35 °C	85 ± 5 a	nd
40 °C	82 ± 3 a	nd
Levantinka	*p* ≤ 0.001	*p* = 0.433
Control	273 ± 6 a	12 ± 1
15 °C	237 ± 4 cd	11 ± 1
20 °C	232 ± 13 d	11 ± 0
25 °C	264 ± 9 ab	11 ± 1
30 °C	258 ± 8 abc	11 ± 0
35 °C	248 ± 8 bcd	11 ± 0
40 °C	232 ± 6 d	11 ± 0
Oblica	*p* ≤ 0.001	*p* ≤ 0.001
Control	244 ± 7 b	11 ± 0 a
15 °C	292 ± 3 a	9 ± 0 ab
20 °C	213 ± 36 bc	7 ± 1 cd
25 °C	221 ± 2 bc	8 ± 0 bc
30 °C	211 ± 1 bc	7 ± 1 cd
35 °C	185 ± 1 c	6 ± 0 d
40 °C	187 ± 2 c	6 ± 0 d

nd—not detected; * variety had a significant effect (*p* ≤ 0.05); ** no variance between results and *p*-value not computed; presented *p*-values indicate the effect of temperature within each variety. Where significant (*p* ≤ 0.05), Tukey’s multiple comparison test was conducted and different letters within column subsections indicate significant differences. All values are mean ± standard deviation of three consecutive production batches.

**Table 5:** Summary of response surface methodology (RSM) model analysis, including *p*-values for the model and lack of fit, coefficient of determination (R^2^), optimal temperature, overall desirability score, and predicted response values for induction period (IP), antioxidant capacity (AC) and volatiles from the lipoxygenase pathway (Σ LOX), oxidation (Σ OX) and microbial activity (Σ MBA).

Variety			Parameters for Optimization				
			IP min	AC (% DPPH Red.)	Σ LOX mg/kg	Σ OX mg/kg	Σ MBA mg/kg
	Model *		y = a + b × T + c × T^2^ + d × T^3^				
Istarska Bjelica *	a		237.017	54.088	5.761	−1.755	−0.593
	b		−3.128	0.484	1.549	0.264	0.059
	c		0.065	−0.009	0.029	−0.004	−0.001
	*p*-Value	Model	0.035	0.362	0.210	0.146	0.040
		Lack of fit	0.056	0.338	0.001	0.007	<0.0001
	R^2^		0.361	0.127	0.189	0.227	0.350
	Optimal temperature		18.9				
	Desirability		0.481				
	Predicted values		201	60.80	24.56	1.51	0.07
Levantinka *	a		119.932	62.511	39.282	3.009	−0.133
	b		2.705	−1.096	0.249	0.004	0.012
	c		−0.047	0.021	−0.005	−2.98 × 10^−5^	−1.54 × 10^−4^
	*p*-Value	Model	0.641	0.107	0.787	0.976	0.027
		Lack of fit	0.209	≤0.0001	0.138	0.458	0.924
		R^2^	0.058	0.258	0.031	0.003	0.383
	Optimal temperature		15.4				
	Desirability		0.518				
	Predicted values		150	50.61	41.75	3.07	0.01
Oblica	a		50.898	1.54	48.997	7.564	−1.316
	b		10.48	7.245	−1.191	−0.141	0.179
	c		−0.489	−0.276	0.015	−0.001	−0.007
	d		0.006	0.003	0 **	0 **	7.99 × 10^−5^
	*p*-Value	Model	≤0.0001	≤ 0.0001	≤0.0001	≤0.0001	0.001
		Lack of fit	0.513	0.001	0.110	0.764	0.001
		R^2^	0.908	0.864	0.772	0.736	0.659
	Optimal temperature		15.5				
	Desirability		0.639				
	Predicted values		119	58.96	34.15	5.65	0.01

* The models obtained for this variety are second-order polynomial regression models; ** The coefficient of d is 0, making it a second-order polynomial model.

## Data Availability

The original contributions generated for this study are included in the article/Supplementary Material; further inquiries can be directed to the corresponding author.

## Supplementary Materials

The following supporting information can be downloaded at: https://www.mdpi.com/article/10.3390/foods14152564/s1, Table S1: Relative retention times (Rt_R_—retention time of the compound in relation to the retention time of syringic acid) for the standards of phenolic compounds detected by UHPLC and HPLC methods; Table S2: Phenolic compounds of virgin olive oil extracts detected and identified by UHPLC Q-TOF-MS and their calculated and experimentally determined relative retention times via the HPLC- DAD method; Table S3: Composition of volatile compounds (VOC), Part I—compounds resulting from lipoxygenase pathway (LOX path) of controls and oils produced with flash thermal treatment; Table S4: Composition of volatile compounds (VOC), Part II—compounds resulting from oxidation (OX) and Part III—compounds resulting from microbiological activities (MBA) of controls and oils produced with flash thermal treatment; Table S5: Fatty acid composition of controls and oils produced with flash thermal treatment. References [104,105,106,107,108] are cited in the Supplementary Materials.
