# Supplementary material for: Improvement of Oxidative Stability and Antioxidative Capacity of Virgin Olive Oil by Flash Thermal Pretreatment—Optimization Process
Source: Foods. 2025 Jul 22;14(15):2564. doi: 10.3390/foods14152564 (PMC12346424; doi:10.3390/foods14152564)
Supplement: Supplementary file 1 [file foods-14-02564-s001.zip › foods-3732554-supplementary.pdf]

Supplementary Material

Improvement of oxidative stability and antioxidative capacity of virgin olive oil by flash thermal pretreatment - Optimization process

Dubravka Škevin [1]*, Sandra Balbino [1], Mirella Žanetić [2,3], Maja Jukić Špika [2,3], Olivera Koprivnjak [4], Katarina Filipan [1], Marko Obranović [1], Karla Žanetić [1], Edina Smajić [1], Mateo Radić [1], Magdalena Bunić [1], Monika Dilber [1] and Klara Kraljić [1]

[1] University of Zagreb Faculty of Food Technology and Biotechnology, Pierottijeva 6, 10000 Zagreb, Croatia;
sandra.balbino@pbf.unizg.hr (S.B.); katarina.filipan@pbf.unizg.hr (K.F.); marko.obranovic@pbf.unizg.hr (M.O.), karla.zanetic.bubi@gmail.com (K.Ž.); smajic.mail@gmail.com (E.S.); mateoradic98@gmail.com (M.R.); mbunic@pbf.hr (M.B.); mdilber@pbf.hr (M.D.); klara.kraljic@pbf.unizg.hr (K.K.)
[2] Institute for Adriatic Crops, Put Duilova 11, 21000 Split, Croatia; Mirella.Zanetic@krs.hr (M.Ž.); Maja.Jukic.Spika@krs.hr (M.J.Š.)
[3] Centre of Excellence for Biodiversity and Molecular Plant Breeding, Svetošimunska Cesta 25, Zagreb, Croatia
[4] University of Rijeka, Faculty of Medicine, Braće Branchetta 20, Rijeka, Croatia; olivera.koprivnjak@medri.uniri.hr (O.K.)
*Correspondence: dubravka.skevin@pbf.unizg.hr (D.Š.)

Table S1. Relative retention times (Rt_R – retention time of the compound in relation to the retention time of syringic acid) for the standards of phenolic compounds detected by UHPLC and HPLC methods.

Phenolic compound	Rt_R (min)	
	UHPLC method	HPLC method
Gallic acid	0.437	0.414
Protocatechuic acid	0.716	0.656
Tyrosol	0.851	0.809
Syringic acid	1.000	1.000
Oleacein	1.202	1.321
Oleuropein	1.242	1.382
Oleocanthal	1.318	1.484
Oleuroside	1.341	1.529
Ligstroside	1.342	1.522
Luteolin	1.489	1.740
Apigenin	1.581	1.908

A very good correlation of the relative retention times for the commercial standards (retention time of each phenolic compound in relation to the retention time of the internal standard - syringic acid) was found between two methods used (UHPLC and HPLC). The best fitting model was the linear regression model represented by Equation (S1)

$$Rt_{R_{\mathrm{HPLC}}}(\mathrm{min}) = 1{,}3387 \times Rt_{R_{\mathrm{UHPLC}}}(\mathrm{min}) - 0{,}2724 \quad \text{(S1)}$$

with a coefficient of determination of R^2=0.9893.

Table S2. Phenolic compounds of virgin olive oil extracts detected and identified by UHPLC Q-TOF-MS and their calculated and experimentally determined relative retention times by HPLC- DAD method.

Phenolic compound	Rt_R* UHPLC (min)	[M-H]⁻ *m/z*	Main fragments *m/z*	Rt_R HPLC (min)		Ref.
				predicted**	experimental	
Hydroxytyrosol[b]	0.68	153.0569	123.0459	0.59	0.61	[104]
Tyrosol[b]	0.85	137.0605	-	0.81	0.8	[105]
Hydroxydecarboxymethyl oleuropein aglycone[a]	0.97	335.1144	151.0406<br>123.045<br>69.0349<br>59.0143	0.97	0.96	[106]
Hydroxy-oleuropein aglycone[a]	i) 1.03<br>ii) 1.04	393.1195	-	i) 1.04<br>ii) 1.06	i) 1.04<br>ii) 1.07	[105]
p-Coumaric acid[a]	1.11	163.0415	119.051	1.14	1.17	[104]
Oleacein[b]	1.21	319.1223	183.0674<br>165.0567<br>69.0351<br>59.0147	1.26	1.30	[106]
Methyl hemiacetal of oleocanthal[a]	1.22	335.1173	111.0818<br>95.0504<br>69.0349<br>59.0143	1.28	1.33	[107]

Oleuropein aglycone[a§]	i) 1.23<br>ii) 1.25<br>iii) 1.41<br>iv) 1.45<br>v) 1.48<br>vi) 1.51	377.1295	307.0856<br>275.0948<br>149.0253<br>95.0507	i) 1.29<br>ii) 1.31<br>iii) 1.51<br>iv) 1.57<br>v) 1.61<br>vi) 1.64	i) 1.34<br>ii) 1.36<br>iii) 1.55<br>iv) 1.64<br>v) 1.67<br>vi) 1.71	[105,106]
Ligstroside aglycone[a£]	i) 1.31<br>ii) 1.31<br>iii) 1.35<br>iv)1.37	361.1328	291.0897<br>259.0994<br>127.0404<br>101.0249	i) + ii) 1.39<br>iii) 1.44<br>iv) 1.46	i) + ii) 1.41<br>iii) 1.50<br>iv) 1.52	[106]
Oleocanthal[b]	1.32	303.1241	179.0737<br>165.0572	1.41	1.48	[106]
Apigenin[b]	1.59	269.04894	-	1.74	1.82	[108]

*Rt_R = relative retention time - retention time of the compound in relation to the retention time of the internal standard - syringic acid); **prediction based on the model expressed by equation (S1); [a]identification based on accurate mass and literature data; [b] identification also confirmed using the standard; [§]no differences were found among MS/MS spectra obtained at six different RRTs. The spectra correspond to those previously reported for oleuropein aglycones, monoaldehydic form of oleuropein aglycone and dialdehydic form of oleuropein aglycone; [£]no differences were found among MS/MS spectra obtained at four different RRTs. The spectra correspond to those previously reported for ligstroside aglycone, monoaldehydic form of ligstroside aglycone, dialdehydic form of ligstroside aglycone and oleokoronal.

Table S3. Composition of volatile compounds (VOC) - Part I – compounds resulting from lipoxygenase pathway (LOX path) of controls and oils produced with flash thermal treatment.

Sample	VOC from LOX path (mg/kg)							
	2-pentenal*	3-hexenal*	2-methyl-4-pentenal*	2-hexenal*	1-penten-3-ol	(E)2-penten-1-ol	(Z)2-penten-1-ol	3-hexen-1-ol
	A	B	B	A	A	B	B	A
Istarska bjelica	*p = 0.205*	*p ≤ 0.01*	**	*p ≤ 0.001*	*p = 0.459*	*p ≤ 0.05*	*p ≤ 0.001*	*p ≤ 0.01*
Control	0.20 ± 0.03	2.84 ± 0.18 a	nd	6.77 ± 0.10 b	1.88 ± 0.16	0.18 ± 0.01 ab	1.81 ± 0.13 ab	1.55 ± 0.23 ab
15 °C	0.12 ± 0.02	2.24 ± 0.15 ab	nd	5.28 ± 0.78bc	4.24 ± 4.65	0.12 ± 0.02 b	1.41 ± 0.17 bc	1.10 ± 0.17 ab
20 °C	0.14 ± 0.06	1.85 ± 0.50 b	nd	4.06 ± 0.83 c	1.42 ± 0.31	0.12 ± 0.03 b	1.23 ± 0.29 c	0.95 ± 0.16 b
25 °C	0.17 ± 0.07	2.23 ± 0.25 ab	nd	11.50 ± 1.50 a	1.96 ± 0.12	0.21 ± 0.07 a	1.85 ± 0.10 a	1.76 ± 0.53 a
30 °C	0.20 ± 0.00	2.21 ± 0.11 ab	nd	6.36 ± 0.22 b	1.67 ± 0.02	0.16 ± 0.01 ab	1.61 ± 0.05 abc	1.42 ± 0.15 ab
35 °C	0.18 ± 0.03	1.94 ± 0.16 b	nd	4.75 ± 0.52 c	1.42 ± 0.09	0.14 ± 0.02 ab	1.30 ± 0.12 c	0.91 ± 0.11 b
40 °C	0.19 ± 0.02	2.35 ± 0.10 ab	nd	5.87 ± 0.68 bc	1.32 ± 0.08	0.13 ± 0.01 ab	1.26 ± 0.05 c	1.21 ± 0.05 ab
Levantinka	*p = 0.359*	*p = 0.580*	*p ≤ 0.05*	*p = 0.421*	*p = 0.061*	*p = 0.311*	*p = 0.076*	*p = 0.687*
Control	0.39 ± 0.01	5.33 ± 1.27	2.41 ± 0.18 a	23.91 ± 0.72	1.50 ± 0.05	0.14 ± 0.01	1.52 ± 0.02	1.84 ± 0.51
15 °C	0.41 ± 0.11	7.85 ± 3.15	2.14 ± 0.70 ab	23.45 ± 0.77	1.40 ± 0.07	0.13 ± 0.01	1.58 ± 0.04	1.62 ± 0.89
20 °C	0.23 ± 0.12	7.58 ± 3.72	0.40 ± 0.66 b	20.25 ± 1.61	1.41 ± 0.16	0.24 ± 0.22	1.52 ± 0.10	1.54 ± 1.56
25 °C	0.27 ± 0.23	5.85 ± 1.32	2.02 ± 1.08 ab	21.40 ± 6.45	1.51 ± 0.08	0.19 ± 0.08	1.67 ± 0.15	5.12 ± 6.64
30 °C	0.39 ± 0.10	5.97 ± 0.42	2.30 ± 0.20 a	23.91 ± 0.58	1.50 ± 0.09	0.13 ± 0.00	1.53 ± 0.09	1.86 ± 0.60
35 °C	0.23 ± 0.13	4.92 ± 1.56	1.28 ± 0.98 ab	20.59 ± 3.59	1.60 ± 0.09	0.30 ± 0.14	1.53 ± 0.03	2.00 ± 1.69
40 °C	0.31 ± 0.07	6.11 ± 1.12	2.10 ± 0.07 ab	19.94 ± 1.79	1.31 ± 0.12	0.12 ± 0.02	1.40 ± 0.10	2.41 ± 0.38
Oblica	*p = 0.141*	*p ≤ 0.05*	*p = 0.075*	*p ≤ 0.05*	*p = 0.075*	*p = 0.503*	*p = 0.335*	*p = 0.301*
Control	0.38 ± 0.07	8.07 ± 2.32 ab	3.41 ± 0.95	7.77 ± 1.81 a	1.42 ± 0.25	0.14 ± 0.04	1.30 ± 0.07	2.32 ± 0.9
15 °C	0.34 ± 0.04	12.45 ± 1.38 a	3.25 ± 0.10	9.98 ± 0.28 b	1.59 ± 0.07	0.12 ± 0.01	1.44 ± 0.02	1.30 ± 0.11
20 °C	0.32 ± 0.07	9.19 ± 3.14 ab	2.37 ± 0.95	7.42 ± 0.58 ab	1.62 ± 0.11	0.35 ± 0.39	2.80 ± 2.27	1.64 ± 0.49
25 °C	0.23 ± 0.11	7.00 ± 2.17 b	2.89 ± 0.28	7.80 ± 1.62 b	1.40 ± 0.04	0.12 ± 0.02	1.29 ± 0.06	4.41 ± 4.44
30 °C	0.34 ± 0.04	6.52 ± 1.19 b	2.31 ± 0.15	7.00 ± 0.14 b	1.48 ± 0.12	0.13 ± 0.00	1.30 ± 0.04	1.57 ± 0.23
35 °C	0.32 ± 0.04	8.08 ± 0.53 ab	2.44 ± 0.08	7.10 ± 0.29 b	1.42 ± 0.03	0.15 ± 0.01	1.40 ± 0.02	1.32 ± 0.38
40 °C	0.38 ± 0.03	6.87 ± 0.58 b	2.22 ± 0.24	6.53 ± 0.73 ab	1.31 ± 0.08	0.14 ± 0.01	1.27 ± 0.09	1.04 ± 0.12

Capital letters below to the compound name indicate the reliability of identification (RID): A-level — agreement of retention index (RI) and mass spectrum with those of an authentic compound; B-level — difference between experimental RI and literature RI < 20 and mass spectrum similarity match > 900; C-level — at least agreement of mass spectrum > 800; nd – not detected; *variety had a significant effect ($p \leq 0.05$); **no variance between results and p-value not computed; presented p-values indicate the effect of temperature within each variety. Where significant ($p \leq 0.05$), Tukey's

multiple comparison test was conducted and different letters within column subsections indicate significant differences. All values are mean ± standard deviation of three consecutive production batches.

Table S3. continuation

Sample	VOC from LOX path (mg/kg)						
	2-hexen-1-ol<br>A	1-hexanol*<br>B	1-pente-3-one*<br>A	hexyl acetate*<br>B	3-hexenyl acetate*<br>A	2-hexenyl acetate*<br>A	Σ LOX*
Istarska bjelica	*p = 0.059*	*p = 0.438*	*p = 0.066*	*p ≤ 0.05*	*p = 0.062*	*p ≤ 0.001*	*p ≤ 0.001*
Control	0.10 ± 0.01	0.48 ± 0.02	5.78 ± 0.34	1.99 ± 0.12 a	5.29 ± 1.80	0.13 ± 0.01 a	28.99 ± 2.19 ab
15 °C	0.08 ± 0.01	0.35 ± 0.03	2.60 ± 2.22	1.57 ± 0.15 ab	4.90 ± 0.31	0.05 ± 0.05 bc	24.05 ± 2.69 bcd
20 °C	0.08 ± 0.03	0.31 ± 0.08	4.39 ± 0.93	1.45 ± 0.46 ab	3.71 ± 1.27	0.01 ± 0.02 c	19.70 ± 4.87 d
25 °C	0.05 ± 0.05	1.11 ± 1.25	5.13 ± 1.34	1.66 ± 0.01 ab	3.34 ± 0.12	nd c	30.96 ± 1.40 a
30 °C	0.11 ± 0.00	0.46 ± 0.02	5.12 ± 0.23	1.81 ± 0.06 ab	5.44 ± 0.36	0.10 ± 0.01 ab	26.66 ± 0.64 abc
35 °C	0.09 ± 0.01	0.35 ± 0.04	4.80 ± 0.42	1.52 ± 0.21 ab	4.06 ± 1.03	0.05 ± 0.02 bc	21.50 ± 1.71 cd
40 °C	0.09 ± 0.01	0.38 ± 0.05	4.67 ± 0.38	1.37 ± 0.12 b	3.34 ± 0.52	0.02 ± 0.02 c	22.21 ± 1.09 bcd
Levantinka	*p ≤ 0.05*	*p ≤ 0.05*	*p = 0.368*	*p = 0.135*	*p = 0.673*	*p = 0.436*	*p = 0.270*
Control	0.04 ± 0.03 a	0.02 ± 0.03 a	4.54 ± 0.10	0.09 ± 0.01	0.24 ± 0.07	0.14 ± 0.25	42.10 ± 0.23
15 °C	0.03 ± 0.03 a	0.09 ± 0.01 a	4.37 ± 0.18	0.10 ± 0.02	0.94 ± 1.16	nd	44.10 ± 2.01
20 °C	0.06 ± 0.05 a	0.10 ± 0.08 a	3.83 ± 0.61	0.13 ± 0.02	0.31 ± 0.06	nd	37.58 ± 7.57
25 °C	0.04 ± 0.04 a	0.06 ± 0.05 a	3.63 ± 1.29	0.11 ± 0.04	0.30 ± 0.09	nd	42.16 ± 4.26
30 °C	0.07 ± 0.04 a	0.07 ± 0.01 a	4.50 ± 0.29	0.06 ± 0.03	0.83 ± 1.16	nd	43.11 ± 1.15
35 °C	5.70 ± 5.08 a	0.60 ± 0.51 a	3.44 ± 1.01	0.10 ± 0.01	0.25 ± 0.08	nd	42.53 ± 1.06
40 °C	0.10 ± 0.02 a	0.11 ± 0.03 a	4.16 ± 0.49	0.12 ± 0.05	0.32 ± 0.12	nd	38.51 ± 2.14
Oblica	*p = 0.463*	*p = 0.522*	*p ≤ 0.05*	**	**	**	*p ≤ 0.05*
Control	nd	nd	4.92 ± 0.4 a	nd	nd	nd	29.73 ± 6.52 ab
15 °C	nd	nd	4.56 ± 0.18 a	nd	nd	nd	35.01 ± 1.34 a
20 °C	nd	nd	4.50 ± 0.25 a	nd	nd	nd	30.21 ± 2.52 ab
25 °C	0.34 ± 0.59	0.43 ± 0.74	3.44 ± 1.21 a	nd	nd	nd	29.36 ± 2.46 ab
30 °C	nd	0.05 ± 0.05	4.83 ± 0.17 a	nd	nd	nd	25.53 ± 1.23 b
35 °C	nd	0.05 ± 0.04	5.25 ± 0.29 a	nd	nd	nd	27.53 ± 0.44 b
40 °C	nd	0.05 ± 0.04	4.86 ± 0.34 a	nd	nd	nd	24.66 ± 1.15 b

Capital letters below to the compound name indicate the reliability of identification (RID): A-level — agreement of retention index (RI) and mass spectrum with those of an authentic compound; B-level — difference between experimental RI and literature RI < 20 and mass spectrum similarity match > 900; C-level — at least agreement of mass spectrum > 800; nd – not detected; *variety had a significant effect (p ≤ 0.05); **no variance between results and p-value not computed; presented p-values indicate the effect of temperature within each variety. Where significant (p ≤ 0.05), Tukey's multiple comparison test was conducted and different letters within column subsections indicate significant differences. All values are mean ± standard deviation of three consecutive production batches.

Table S4. Composition of volatile compounds (VOC) - Part II – compounds resulting from oxidation (OX) and Part III - compounds resulting from microbiological activities (MBA) of controls and oils produced with flash thermal treatment.

Sample	VOC from OX (mg/kg)				VOC from MBA (mg/kg)		
	pentanal<br>A	2,4-hexadienal*<br>A	nonanal*<br>A	Σ OX*	2-methylbutanal*<br>C	3-methylbutanal*<br>B	Σ MBA*
Istarska bjelica	*p ≤ 0.01*	*p = 0.312*	*p ≤ 0.05*	*p ≤ 0.01*	*p ≤ 0.001*	*p ≤ 0.001*	*p ≤ 0.001*
Control	0.69 ± 0.24 b	0.75 ± 0.06	0.15 ± 0.02 ab	1.58 ± 0.19 a	0.04 ± 0.04 bc	0.08 ± 0.01 bc	0.12 ± 0.05 bc
15 °C	0.36 ± 0.02 b	0.65 ± 0.04	0.13 ± 0.01 ab	1.14 ± 0.05 a	0.01 ± 0.01 c	0.05 ± 0.01 c	0.06 ± 0.02 c
20 °C	0.54 ± 0.05 a	0.49 ± 0.19	0.17 ± 0.04 ab	1.20 ± 0.27 a	0.04 ± 0.02 bc	0.08 ± 0.02 bc	0.12 ± 0.03 bc
25 °C	1.92 ± 1.03 b	0.65 ± 0.13	0.11 ± 0.01 b	2.67 ± 0.90 a	0.16 ± 0.01 a	0.28 ± 0.01 a	0.44 ± 0.02 a
30 °C	0.58 ± 0.09 b	0.55 ± 0.10	0.20 ± 0.04 a	1.34 ± 0.23 a	0.07 ± 0.00 b	0.11 ± 0.01 b	0.18 ± 0.01 b
35 °C	0.70 ± 0.09 b	0.51 ± 0.21	0.16 ± 0.01 ab	1.37 ± 0.28 a	0.07 ± 0.01 b	0.11 ± 0.02 b	0.17 ± 0.03 b
40 °C	0.49 ± 0.22 b	0.56 ± 0.14	0.19 ± 0.03 a	1.23 ± 0.21 a	0.08 ± 0.01 b	0.13 ± 0.01 b	0.21 ± 0.02 b
Levantinka	*p = 0.361*	*p = 0.428*	*p ≤ 0.01*	*p = 0.686*	*p = 0.133*	*p = 0.139*	*p = 0.119*
Control	0.51 ± 0.29	2.84 ± 0.41	0.15 ± 0.01 bc	3.49 ± 0.66	0.06 ± 0.01	0.06 ± 0.01	0.12 ± 0.02
15 °C	0.34 ± 0.06	2.41 ± 0.43	0.19 ± 0.02 abc	2.94 ± 0.47	nd	0.01 ± 0.01	0.01 ± 0.01
20 °C	0.79 ± 0.78	2.27 ± 1.22	0.19 ± 0.01 abc	3.25 ± 0.44	0.03 ± 0.03	0.02 ± 0.02	0.05 ± 0.05
25 °C	0.70 ± 0.66	2.38 ± 0.84	0.13 ± 0.01 c	3.21 ± 0.58	0.03 ± 0.03	0.04 ± 0.04	0.07 ± 0.06
30 °C	0.35 ± 0.07	2.62 ± 0.20	0.18 ± 0.02 abc	3.15 ± 0.15	0.04 ± 0.03	0.06 ± 0.02	0.09 ± 0.06
35 °C	1.13 ± 0.60	1.45 ± 1.23	0.20 ± 0.03 ab	2.78 ± 0.72	0.03 ± 0.03	0.05 ± 0.02	0.08 ± 0.05
40 °C	0.39 ± 0.02	2.74 ± 0.35	0.22 ± 0.04 a	3.34 ± 0.36	0.05 ± 0.02	0.06 ± 0.02	0.11 ± 0.04
Oblica	*p = 0.323*	*p ≤ 0.001*	*p = 0.333*	*p ≤ 0.01*	*p ≤ 0.001*	*p ≤ 0.001*	*p ≤ 0.001*
Control	0.24 ± 0.11	5.35 ± 0.92 ab	0.15 ± 0.05	5.73 ± 0.85 a	0.01 ± 0.01 bc	0.03 ± 0.03 bc	0.04 ± 0.03 bc
15 °C	0.17 ± 0.05	5.47 ± 0.39 a	0.13 ± 0.05	5.77 ± 0.39 a	0.04 ± 0.03 abc	0.07 ± 0.03 abc	0.11 ± 0.07 abc
20 °C	0.40 ± 0.13	4.53 ± 0.32 ab	0.19 ± 0.01	5.12 ± 0.44 ab	0.05 ± 0.02 ab	0.07 ± 0.01 ab	0.12 ± 0.03 ab
25 °C	0.62 ± 0.60	3.96 ± 0.92 abc	0.15 ± 0.02	4.73 ± 0.74 ab	0.08 ± 0.01 a	0.12 ± 0.02 c	0.20 ± 0.03 a

30 °C	0.48 ± 0.01	3.65 ± 0.48 bc	0.15 ± 0.02	4.27 ± 0.5 ab	nd c	nd c	nd c
35 °C	0.50 ± 0.04	3.61 ± 0.36 bc	0.15 ± 0.01	4.26 ± 0.36 ab	nd c	nd c	nd c
40 °C	0.47 ± 0.04	3.04 ± 0.26 c	0.12 ± 0.00	3.63 ± 0.24 b	nd c	nd c	nd c

Capital letters below to the compound name indicate the reliability of identification (RID): A-level — agreement of retention index (RI) and mass spectrum with those of an authentic compound; B-level — difference between experimental RI and literature RI < 20 and mass spectrum similarity match > 900; C-level — at least agreement of mass spectrum > 800; nd – not detected; *variety had a significant effect ($p \leq 0.05$); presented p-values indicate the effect of temperature within each variety. Where significant ($p \leq 0.05$), Tukey's multiple comparison test was conducted and different letters within column subsections indicate significant differences. All values are mean ± standard deviation of three consecutive production batches.
.

Table S5. Fatty acid composition of controls and oils produced with flash thermal treatment

Sample	Fatty acid (% of total)									
	C16:0*	C16:1*	C18:0*	C18:1*	C18:2*	C18:3*	C20:0*	C20:1	Σ SFA*	Σ MUFA*	Σ PUFA*
Istarska bjelica	*p ≤ 0.05*	*p ≤ 0.01*	*p ≤ 0.05*	*p ≤ 0.001*	*p = 0.123*	**	*p ≤ 0.05*	**	*p ≤ 0.01*	*p ≤ 0.001*	*p = 0.123*
Control	13.1 ± 0.2 ab	1.2 ± 0.1 a	3.3 ± 0.1 a	72.4 ± 0.6 c	5.9 ± 0.1	0.4 ± 0.0	0.4 ± 0.1 a	0.2 ± 0.0	16.8 ± 0.1 ab	73.8 ± 0.5 c	6.3 ± 0.1
15	12.9 ± 0.1 b	1.1 ± 0.0 a	3.4 ± 0.0 a	73.7 ± 0.1 a	6.0 ± 0.0	0.4 ± 0.0	0.5 ± 0.0 a	0.2 ± 0.0	16.8 ± 0.1 b	75.0 ± 0.1 a	6.4 ± 0.0
20	13.1 ± 0.1 ab	1.2 ± 0.0 a	3.4 ± 0.1 a	73.1 ± 0.3 abc	5.9 ± 0.0	0.4 ± 0.0	0.5 ± 0.0 a	0.2 ± 0.0	17.0 ± 0.1 ab	74.5 ± 0.3 ab	6.3 ± 0.0
25	13.1 ± 0.1 a	1.2 ± 0.0 a	3.4 ± 0.1 a	72.8 ± 0.3 bc	6.0 ± 0.0	0.4 ± 0.0	0.5 ± 0.0 a	0.2 ± 0.0	17.0 ± 0.0 a	74.2 ± 0.3 bc	6.4 ± 0.0
30	13.0 ± 0.1 ab	1.2 ± 0.0 a	3.4 ± 0.0 a	73.5 ± 0.1 ab	6.0 ± 0.0	0.4 ± 0.0	0.5 ± 0.0 a	0.2 ± 0.0	16.9 ± 0.1 ab	74.9 ± 0.1 ab	6.4 ± 0.0
35	13.1 ± 0.1 ab	1.2 ± 0.0 a	3.4 ± 0.0 a	73.4 ± 0.2 ab	6.0 ± 0.1	0.4 ± 0.0	0.5 ± 0.0 a	0.2 ± 0.0	17.0 ± 0.1 ab	74.8 ± 0.2 ab	6.4 ± 0.1
40	13.1 ± 0.0 ab	1.2 ± 0.0 a	3.4 ± 0.1 a	73.2 ± 0.2 ab	6.0 ± 0.1	0.4 ± 0.0	0.5 ± 0.0 a	0.2 ± 0.0	17.0 ± 0.1 ab	74.6 ± 0.2 ab	6.4 ± 0.1
Levantinka	*p ≤ 0.05*	*p ≤ 0.01*	*p = 0.393*	*p = 0.082*	*p ≤ 0.05*	**	**	**	*p ≤ 0.01*	*p = 0.128*	*p ≤ 0.05*
Control	11.4 ± 0.1 b	0.5 ± 0.0 b	3.0 ± 0.1	76.7 ± 0.1	5.3 ± 0.1 b	0.6 ± 0.0	0.5 ± 0.0	0.3 ± 0.0	14.9 ± 0.0 b	77.5 ± 0.1	5.9 ± 0.1 b
15	11.6 ± 0.1 ab	0.6 ± 0.0 a	3.0 ± 0.1	76.2 ± 0.3	5.5 ± 0.1 ab	0.6 ± 0.0	0.5 ± 0.0	0.3 ± 0.0	15.0 ± 0.1 ab	77.1 ± 0.3	6.1 ± 0.1 ab
20	11.7 ± 0.1 a	0.6 ± 0.0 a	3.0 ± 0.1	76.0 ± 0.3	5.6 ± 0.1 a	0.6 ± 0.0	0.5 ± 0.0	0.3 ± 0.0	15.2 ± 0.1 a	76.9 ± 0.3	6.2 ± 0.1 a
25	11.5 ± 0.0 ab	0.6 ± 0.0 a	3.0 ± 0.1	76.3 ± 0.2	5.4 ± 0.1 ab	0.6 ± 0.0	0.5 ± 0.0	0.3 ± 0.0	15.0 ± 0.1 ab	77.2 ± 0.2	6.0 ± 0.1 ab
30	11.6 ± 0.1 ab	0.6 ± 0.1 ab	3.0 ± 0.0	76.5 ± 0.4	5.3 ± 0.2 ab	0.6 ± 0.0	0.5 ± 0.0	0.3 ± 0.0	15.1 ± 0.1 a	77.3 ± 0.4	5.9 ± 0.2 ab
35	11.6 ± 0.2 ab	0.5 ± 0.1 ab	3.0 ± 0.1	76.5 ± 0.4	5.5 ± 0.2 ab	0.6 ± 0.0	0.5 ± 0.0	0.3 ± 0.0	15.0 ± 0.2 ab	77.3 ± 0.4	6.1 ± 0.2 ab
40	11.7 ± 0.1 a	0.6 ± 0.0 a	3.0 ± 0.0	76.2 ± 0.2	5.6 ± 0.1 a	0.6 ± 0.0	0.5 ± 0.0	0.3 ± 0.0	15.2 ± 0.1 a	77.1 ± 0.2	6.2 ± 0.1 a
Oblica	*p ≤ 0.001*	**	*p = 0.361*	*p ≤ 0.001*	*p = 0.350*	**	*p ≤ 0.001*	**	*p ≤ 0.05*	*p ≤ 0.01*	*p = 0.535*
Control	13.5 ± 0.1 b	0.7 ± 0.0	2.5 ± 0.0	69.2 ± 0.2 ab	11.4 ± 0.2	0.6 ± 0.0	0.5 ± 0.0 a	0.3 ± 0.0	16.5 ± 0.1 ab	70.2 ± 0.2 abc	12 ± 0.2
15	13.9 ± 0.1 a	0.8 ± 0.0	2.4 ± 0.1	68.7 ± 0.2 b	11.4 ± 0.1	0.7 ± 0.0	0.5 ± 0.0 a	0.3 ± 0.0	16.8 ± 0.0 a	69.8 ± 0.2 c	12.1 ± 0.1

20	13.3 ± 0.1 bc	0.7 ± 0.0	2.7 ± 0.1	69.5 ± 0.5 a	11.3 ± 0.4	0.7 ± 0.0	0.5 ± 0.0 a	0.3 ± 0.0	16.5 ± 0.1 ab	70.5 ± 0.5 ab	12.0 ± 0.4
25	13.8 ± 0.0 a	0.8 ± 0.0	2.5 ± 0.0	68.8 ± 0.1 b	11.5 ± 0.0	0.7 ± 0.0	0.5 ± 0.0 a	0.3 ± 0.0	16.8 ± 0.0 a	69.9 ± 0.1 bc	12.2 ± 0.0
30	13.1 ± 0.1 c	0.7 ± 0.0	1.7 ± 1.2	69.7 ± 0.2 a	11.5 ± 0.1	0.6 ± 0.0	0.5 ± 0.0 a	0.3 ± 0.0	15.3 ± 1.2 b	70.7 ± 0.2 a	12.1 ± 0.1
35	13.1 ± 0.0 c	0.7 ± 0.0	2.4 ± 0.0	69.3 ± 0.1 ab	11.5 ± 0.0	0.6 ± 0.0	0.4 ± 0.1 ab	0.3 ± 0.0	15.9 ± 0.1 ab	70.3 ± 0.1 abc	12.1 ± 0.0
40	13.1 ± 0.0 c	0.7 ± 0.0	2.4 ± 0.0	69.1 ± 0.1 ab	11.6 ± 0.1	0.6 ± 0.0	0.4 ± 0.0 b	0.3 ± 0.0	15.9 ± 0.0 ab	70.1 ± 0.1 abc	12.2 ± 0.1

*Variety had a significant effect ($p \leq 0.05$); **no variance between results and p-value not computed; presented p-values indicate the effect of temperature within each variety. Where significant ($p \leq 0.05$), Tukey's multiple comparison test was conducted and different letters within column subsections indicate significant differences. All values are mean ± standard deviation of three consecutive production batches.

.

References

104. Quirantes-Piné, R.; Lozano-Sánchez, J.; Herrero, M.; Ibáñez, E.; Segura-Carretero, A.; Fernández-Gutiérrez, A. HPLC–ESI–QTOF–MS as a Powerful Analytical Tool for Characterising Phenolic Compounds in Olive-leaf Extracts. Phytochem Anal 2013, 24, 213–223, doi:10.1002/pca.2401.
105. Kalogiouri, N.P.; Kritikou, E.; Martakos, I.C.; Lazarou, C.; Pentogennis, M.; Thomaidis, N.S. Characterization of the Phenolic Fingerprint of Kolovi Extra Virgin Olive Oils from Lesvos with Regard to Altitude and Farming System Analyzed by UHPLC-QTOF-MS. Molecules 2021, 26, 5634, doi:10.3390/molecules26185634.
106. Kanakis, P.; Termentzi, A.; Michel, T.; Gikas, E.; Halabalaki, M.; Skaltsounis, A.-L. From Olive Drupes to Olive Oil. An HPLC-Orbitrap-Based Qualitative and Quantitative Exploration of Olive Key Metabolites. Planta Med 2013, 79, 1576–1587, doi:10.1055/s-0033-1350823.
107. Sánchez de Medina, V.; Miho, H.; Melliou, E.; Magiatis, P.; Priego-Capote, F.; Luque de Castro, M.D. Quantitative Method for Determination of Oleocanthal and Oleacein in Virgin Olive Oils by Liquid Chromatography–Tandem Mass Spectrometry. Talanta 2017, 162, 24–31, doi:10.1016/j.talanta.2016.09.056.
108. Jerman Klen, T.; Golc Wondra, A.; Vrhovšek, U.; Mozetič Vodopivec, B. Phenolic Profiling of Olives and Olive Oil Process-Derived Matrices Using UPLC-DAD-ESI-QTOF-HRMS Analysis. J Agric Food Chem 2015, 63, 3859–3872, doi:10.1021/jf506345q.
